# Genomic View of Bipolar Disorder Revealed by Whole Genome Sequencing in a Genetic Isolate

**DOI:** 10.1371/journal.pgen.1004229

**Published:** 2014-03-13

**Authors:** Benjamin Georgi, David Craig, Rachel L. Kember, Wencheng Liu, Ingrid Lindquist, Sara Nasser, Christopher Brown, Janice A. Egeland, Steven M. Paul, Maja Bućan

**Affiliations:** 1 Department of Genetics, Perelman School of Medicine, University of Pennsylvania, Philadelphia, Pennsylvania, United States of America; 2 The Translational Genomics Research Institute, Phoenix, Arizona, United States of America; 3 Department of Neuroscience, University of Miami Miller School of Medicine, Miami, Florida, United States of America; 4 Department of Psychiatry and Behavioral Sciences, University of Miami Miller School of Medicine, Miami, Florida, United States of America; 5 Department of Pharmacology, Weill Cornell Medical College, New York, New York, United States of America; 6 Department of Psychiatry, Weill Cornell Medical College, New York, New York, United States of America; 7 Department of Psychiatry, Perelman School of Medicine, University of Pennsylvania, Philadelphia, Pennsylvania, United States of America; The Wellcome Trust Centre for Human Genetics, University of Oxford, United Kingdom

## Abstract

Bipolar disorder is a common, heritable mental illness characterized by recurrent episodes of mania and depression. Despite considerable effort to elucidate the genetic underpinnings of bipolar disorder, causative genetic risk factors remain elusive. We conducted a comprehensive genomic analysis of bipolar disorder in a large Old Order Amish pedigree. Microsatellite genotypes and high-density SNP-array genotypes of 388 family members were combined with whole genome sequence data for 50 of these subjects, comprising 18 parent-child trios. This study design permitted evaluation of candidate variants within the context of haplotype structure by resolving the phase in sequenced parent-child trios and by imputation of variants into multiple unsequenced siblings. Non-parametric and parametric linkage analysis of the entire pedigree as well as on smaller clusters of families identified several nominally significant linkage peaks, each of which included dozens of predicted deleterious variants. Close inspection of exonic and regulatory variants in genes under the linkage peaks using family-based association tests revealed additional credible candidate genes for functional studies and further replication in population-based cohorts. However, despite the in-depth genomic characterization of this unique, large and multigenerational pedigree from a genetic isolate, there was no convergence of evidence implicating a particular set of risk loci or common pathways. The striking haplotype and locus heterogeneity we observed has profound implications for the design of studies of bipolar and other related disorders.

## Author Summary

Bipolar disorder is a common, heritable mental illness characterized by recurrent episodes of mania and depression. Despite considerable efforts genetic studies have yet to reveal the precise genetic underpinnings of the disorder. In this study we have analyzed a large extended pedigree of Old Order Amish that segregates bipolar disorder. Our study design integrates both dense genotype and whole-genome sequence data. In a combined linkage and association analysis we identify five chromosomal regions with nominally significant or suggestive evidence for linkage, several of which constitute replication of earlier linkage findings for bipolar disorder in non-Amish families. Association analysis of genetic variants in each of the linkage regions yielded a number of plausible candidate genes for bipolar disorder. The striking genetic heterogeneity we observed in this genetic isolate has profound implications for the study of bipolar disorder in the general population.

## Introduction

Bipolar affective disorder is a life-long mental illness characterized by recurrent episodes of depression and mania or hypomania, with a typical age of onset in young adulthood. Twin and family studies have shown that bipolar disorder has a strong genetic component with heritability estimated to be in the range of 80% [1]. As such, families with bipolar disorder have been extensively studied by linkage analysis [2] and a considerable number of possible susceptibility loci have been reported. The largest linkage study to date on 972 unrelated families identified loci on chromosomes 6q21 and 9q21 [3]. In addition, genome-wide association studies (GWAS) using large cohorts of patients and control subjects have identified single nucleotide polymorphisms (SNPs) in *CACNA1C* (alpha 1C subunit of the L-type voltage-gated calcium channel) and *ANK3* (ankyrin 3) implicating these as potential susceptibility genes for bipolar disorder [4]–[6]. A recent meta-analysis of GWA data from over 13,600 individuals identified a risk locus for major mood disorders at 3p21.1 [7]. A large-scale international GWAS effort replicated the association for *CACNA1C* and identified new susceptibility alleles in *ODZ4* [8]. Although genome-wide association studies remain an important approach for identifying common variants conferring risk for common psychiatric disorders, each individual locus or allele identified to date explains only a very small proportion of familial clustering and relatively small changes in risk [9]–[11].

Genetic studies of isolated populations have led to the identification of genetic variants underlying a number of Mendelian disorders, particularly those caused by autosomal recessive mutations [12], [13]. More recently, homozygosity mapping of rare recessive mutations in consanguineous families has identified mutations and copy number variants (CNVs) in neurodevelopmental disorders, such as autism and hereditary sensory and autonomic neuropathy type IV [14], [15]. Genetically isolated populations, characterized by reduced genetic, phenotypic and environmental heterogeneity, serve as a powerful model for studies of allelic diversity associated with common neurodevelopmental and psychiatric disorders.

The Old Order Amish are a genetic isolate of European ancestry currently residing in several states in North America, with a concentration in Pennsylvania and Ohio [16]. Bipolar disorder type I (BPI) and bipolar disorder type II (BPII) in the Amish occur with similar prevalence, pattern of symptoms, clinical course and response to mood-stabilizing medicines as observed in the general North American population [17]–[19]. Alcohol and drug abuse, which often complicate psychiatric diagnoses, are rare among the Amish. Their lifestyle provides a remarkably uniform environment in which behavioral changes can be readily and longitudinally ascertained.

The original genetic linkage study of a large extended Old Order Amish pedigree with bipolar affective disorder reported positive findings with DNA markers around the *HRAS1* and *INS* loci on chromosome 11 [20]. When the original Amish pedigree was further extended and updated clinical data were incorporated, linkage at the chromosome 11 locus was excluded [21]. An early genome-wide linkage scan using 551 microsatellite markers revealed possible bipolar susceptibility loci on chromosomes 6, 13, and 15, suggesting that bipolar disorder, even in a genetic isolate, is likely inherited as a complex trait [22].

To identify the genetic basis of bipolar disorder in the Amish, our research returned to the expanded multigenerational Old Order Amish pedigree available as the Amish Study of Major Affective Disorders at the Coriell Institute for Medical Research and applied contemporary genomic and statistical methodology by integrating genotype and whole-genome sequence data. This investigation combines linkage analyses on multiallelic microsatellite genotypes for the large pedigree (n = 497) with the analysis of Illumina Omni 2.5M SNP genotypes (n = 388) and whole-genome sequences of 50 family members. Our findings reveal multiple linkage regions that each harbor a considerable number of sequence variants, supporting initially reported locus heterogeneity. Dissection of exonic and intronic variants that reside in these linkage peaks has identified credible candidate genes that will be further examined in large-scale population-based studies. Our results underscore the complexity of the genetic etiology of bipolar disorder in this genetic isolate and have important implications for the study of bipolar disorder in the general population.

## Results

### Genotype and whole genome sequence data

**Figure 1:**
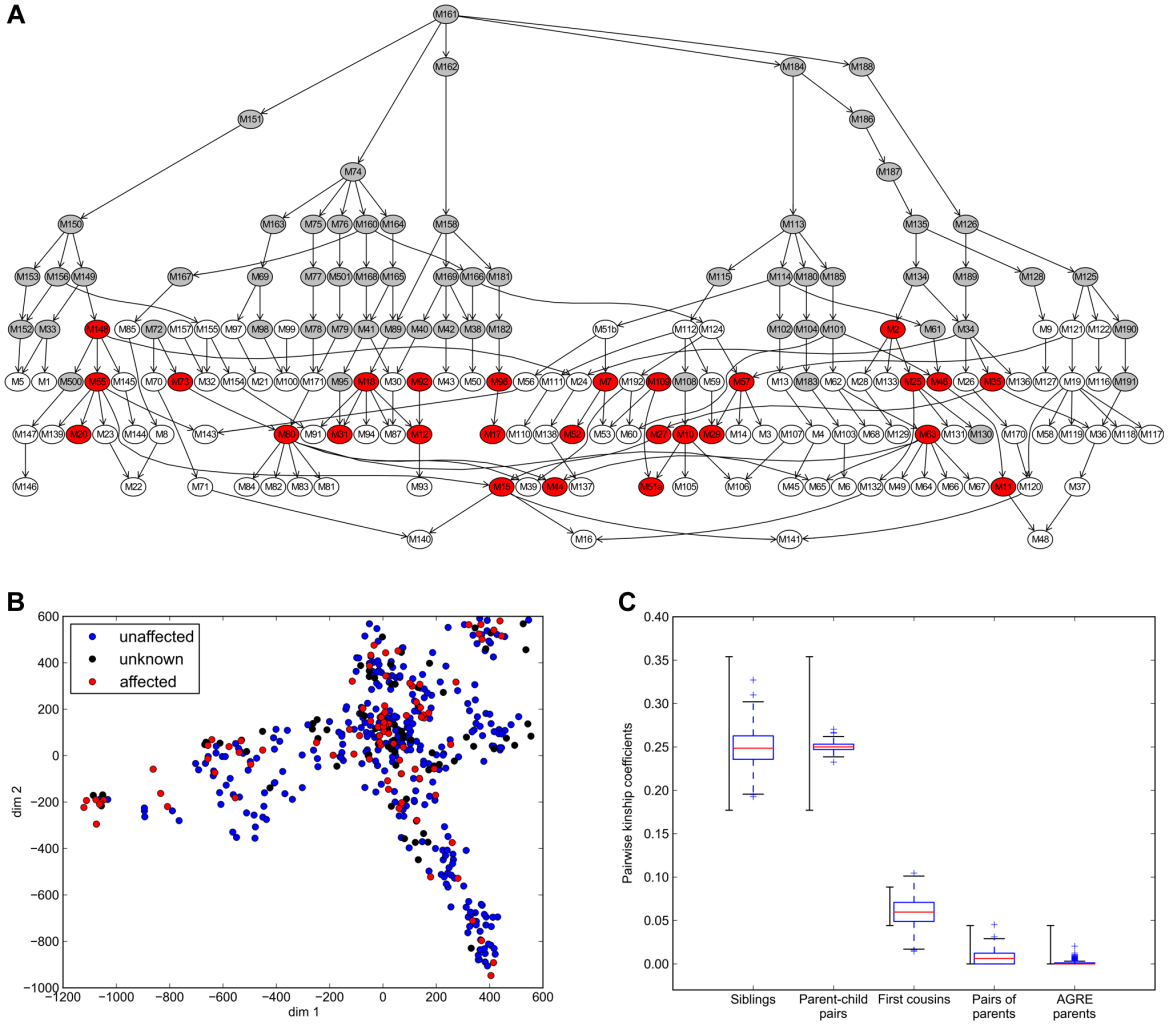
Amish pedigree sub-structure. A) Nuclear family graph representation of the entire pedigree. Each node represents a nuclear family and families are connected by parent-child relationships. The pedigree includes families with genealogical information but without DNA available (grey), families with SNP genotype data available (white and red) and families with at least one WGS subject (red). B) Visualization by multidimensional scaling (MDS) of population substructure and distribution of affection status in the extended pedigree based on microsatellite genotypes. Individuals with Major Affective disorder (BPI or BPII) are shown in red, unaffected individuals in blue. Subjects with other mental illness are depicted as having unknown phenotype (black). C) Pair wise kinship coefficients for known family relationships in the Amish pedigree and an independent set of unrelated parents from the Autism Genetics Research Exchange (AGRE) [88]. As reported in _ENREF_34 [28], expected kinship coefficients are >0.354 for duplicate samples/monozygotic twins, [0.177–0.354] for 1^st^ degree relatives, [0.0884–0.177] for 2^nd^ degree relative, [0.0442–0.0884] for 3^rd^ degree relatives and <0.0442 for unrelated subjects The expected range of kinship coefficients is shown to the left, the observed kinship coefficients to the right.

The Amish Study of Major Affective Disorders includes biomaterials and clinical data for a large extended family (497 family members) (Figures 1A and 1B). The Amish Study Psychiatric Board (see Methods) established clinical diagnoses for each family member using Research Diagnostic Criteria (RDC) [23] and DSM-III/IV criteria [24]. We obtained: a) genotypes from a panel of 1991 multi-allelic microsatellite markers (deCODE panel) for the entire pedigree (497 individuals) and b) high-density SNP genotypes (Illumina Omni 2.5 M SNP arrays) for a subset of 388 individuals. Copy number variants (CNVs) were called using the PennCNV software [25]. To establish an initial catalogue of potential sequence variants underlying bipolar disorder, we also obtained whole genome sequences (WGS) for 50 individuals comprising 18 parent-child trios and 8 additional parent-child pairs from the extended pedigree (Figure S1). We selected individuals for whole genome sequencing that included a) the most distantly related subfamilies; b) affected individuals (n = 23) diagnosed with BPI or BPII and c) a small subset of healthy siblings (ages between 48 and 78, well past the age of disease onset, which is usually prior to age 35). Whole genome sequencing was performed by Complete Genomics Inc. (CGI; Mountain View, CA) using a sequence-by-ligation method [26]. Median sequencing depth was ∼50× across all 50 samples (Figure S2). By combining genotype data for the entire extended pedigree with whole genome sequences for a selected set of parent-child trios across different sub-pedigrees, we were able to infer the genetic background for the entire pedigree and address the impact of both common and rare variants within this large multigenerational family.

### Bipolar disorder phenotypic categories and heritability estimates

In order to address genetic and phenotypic heterogeneity, analysis was restricted to 49 nuclear families with at least one BPI subject. Among the 364 selected subjects were 92 individuals with Major Affective Disorder including bipolar disorder I (BPI) (n = 62), bipolar disorder II (BPII) (n = 15) and Major Depressive Disorder, recurrent form (MDD-R) (n = 15); 52 individuals with minor psychiatric diagnoses (classified as “unknown” phenotype) and 220 “well” subjects. Three diagnostic hierarchies were defined for the analysis: the first and most stringent classification included only BPI individuals as affected (BPI phenotype), the second included BPI and BPII individuals as affected (narrow phenotype) and the third included BPI, BPII and MDD-R as affected (bipolar spectrum disorder or BPS).

To quantify the fraction of phenotypic variance accounted for by genetic causes, we estimated the narrow sense heritability (h^2^) of bipolar susceptibility in the Amish pedigree using the Sequential Oligogenic Linkage Analysis Routines (SOLAR) [27] software. We observed significant heritability for all three phenotype definitions (BPI, narrow, BPS). The most heritable phenotype was BPS with a heritability of 0.626 (P = 0.0000015) (Table 1).

**Table 1:** Estimates of additive heritability for the BPI, Narrow and BPS phenotype definitions.

Phenotype	BPI	Narrow	BPS
h^2^ **heritability estimates (SE)**	0.364 (0.163)	0.526 (0.184)	0.626 (0.194)
**P-value**	0.0023192	0.0000199	0.0000015

All phenotypes show significant heritability.

### Analysis of relatedness in the extended pedigree using high-density genotypes

To quantify the overall degree of relatedness of individuals within the extended pedigree and to provide quality control of our genotype data, pair-wise kinship coefficients were calculated based on the SNP array genotypes using the KING software [28] (Figure S3). Observed ranges of kinship coefficients for known family relationships matched well with expectations and coefficients obtained from an independent dataset of 569 unrelated parent pairs (Figure 1C). To estimate the overall level of consanguinity and identify the most consanguineous nuclear families, we investigated relatedness between 32 pairs of parents. While the overall relatedness of parent pairs in the pedigree is higher than for the control parent pairs, the bulk of the distribution falls into the range of kinship expected for unrelated individuals (kinship coefficients <0.0442). A single, four-member family showed a kinship coefficient of 0.0453 between the parents, which falls into the expected range for third degree relatives. The overall low kinship coefficient estimates between pairs of parents suggests that inbreeding among close relatives is uncommon in this pedigree, and it is unlikely to bias subsequent analyses.

### Initial analysis of whole genome sequence data

The sequencing of 50 Amish genomes identified 11.1 million sequence variants within the 97% called genome fraction. From the ∼3.79 million SNPs and indels detected in each individual, 20,000 fell within exons, with an average of 10,000 synonymous SNPs and roughly the same number of non-synonymous, frame shift or gain/loss of a stop-codon variants. The overall rate of novel SNP variants per sample is ∼1.3% with respect to dbSNP 138. We initially searched for exonic missense variants common in the 23 individuals with BPI/II and rare (MAF<2%) in the 1000 Genomes Project data, the EVS database and a set of 54 HapMap WGS made publicly available by CGI. Of the resulting list of 514 variants predicted to be “damaging/deleterious” by both Polyphen2 [29] and SIFT [30] none were both present in all BP I/II samples and absent or rare in 1000 Genomes data. The same was true when we prioritized exonic variants based on the degree of nucleotide-level evolutionary conservation using Genomic Evolutionary Rate Profiling (GERP) conservation scores ([31]; see Table S1 for the 50 most constrained variants). This gives further support to previously reported genetic analysis [22], which revealed a complex mode of inheritance and genetic heterogeneity of bipolar disorder in this large multigenerational family. Table 2 shows the top 30 putatively damaging missense SNPs enriched among this Amish pedigree and rare in the general population (see Table S2 for the full list of 514 variants).

**Table 2:** Top 30 Amish-specific putative damaging exonic missense variants by prevalence in affected individuals.

Chr.	Position	Ref	Alt	dbSNP 138	*Gene*	#BP1	# Well	1000G freq.	EVS freq.
chr8	39521389	T	C	rs147445686	*ADAM18*	13	8	.	0.0023
chr8	37692704	G	A	rs113270504	*GPR124*	13	9	0.011	0.017
chr6	42146043	A	G	rs200275941	*GUCA1A*	12	7	.	.
chr1	40020020	C	A	rs79473113	*LOC728448*	10	8	.	.
chrX	65835841	A	G	rs73221529	*EDA2R*	10	10	0.0024	0.007
chr19	14164629	C	T	rs75841596	*PALM3*	9	11	0.017	.
chr2	61413761	C	A	rs200746889	*AHSA2*	8	1	.	0.00015
chr8	120430415	G	A	Novel	*NOV*	8	5	.	.
chr9	5164252	C	T	Novel	*INSL6*	8	7	.	.
chr1	1387499	T	A	rs199583997	*ATAD3C*	8	8	.	0.0018
chr18	76335864	G	A	rs149637223	*LOC100132713*	8	10	.	.
chr1	27661952	C	T	rs150461998	*TMEM222*	8	10	0.0096	0.009
chr16	25238420	G	A	rs117255669	*AQP8*	7	1	0.0087	0.017
chr19	9067237	T	G	rs201826382	*MUC16*	7	3	.	.
chr2	96907615	A	C	Novel	*LOC285033*	7	3	.	.
chr16	48204130	C	T	rs60681475	*ABCC11*	7	4	0.0078	0.011
chr6	84925066	A	G	rs147915749	*KIAA1009*	7	4	0.00091	0.003
chr6	84904604	G	C	rs17790493	*KIAA1009*	7	4	0.0179	0.019
chr19	9059827	A	G	rs76869876	*MUC16*	7	4	0.0046	0.006
chr3	172365793	C	T	Novel	*NCEH1*	7	5	.	.
chr11	48267242	G	A	rs145684951	*OR4X2*	7	5	.	0.0002
chr2	163302901	C	T	rs78247304	*KCNH7*	7	5	.	.
chr11	55798414	G	T	rs142890517	*OR5AS1*	7	5	.	7.70E-05
chr17	3195777	A	T	rs149826123	*OR3A1*	7	6	0.0037	0.004
chr7	120911438	G	A	rs138155176	*C7orf58*	7	6	0.00092	0.0017
chr22	26937139	C	T	rs117701840	*TPST2*	7	6	0.0041	0.005
chr12	110566907	A	G	rs34684319	*IFT81*	7	6	0.014	0.0145
chr10	75860740	A	G	rs71579374	*VCL*	7	7	0.00046	0.0009
chr1	1269071	A	G	rs376489341	*TAS1R3*	7	8	.	7.70E-05
chr19	12429782	G	A	rs112896133	*ZNF563*	7	8	0.0041	0.0088

Exonic missense variants, identified in 50 subjects with WGS, were filtered by <2% allele frequency in 1000 Genomes, EVS and 54 HapMap WGS. Functional impact was assessed by consensus of Polyphen2 and SIFT.

### Genome-wide association analysis

To explore the role of common variants in bipolar susceptibility and to prioritize variants under linkage peaks (see below), we performed family based genome-wide association analysis on 388 samples genotyped with Illumina Omni 2.5 Million SNP arrays. We applied two methods for association analysis: (a) EMMAX [32], a mixed model association method accounting for population structure, including pairwise relatedness and (b) FBAT [33], an extension of the classical transmission distortion test (TDT) [34] to larger families. These two methods are complementary, as EMMAX quantifies differences in allele frequency in affected and unaffected subjects while FBAT considers deviations from random segregation of alleles in affected and unaffected children. After conducting quality control of the SNP genotype data (see Methods), 1.3M SNPs were retained for association analysis (see Figure S4 for quantile-quantile plots). We did not observe genome-wide significant association (p<5e-8) with either method (Figure S5). We hypothesized that meaningful associations may be found among SNPs that showed the lowest P values (p< = 5e-4) using both methods (Table S3). The two SNPs with the strongest association (p<5e-5 for both algorithms) were rs13317247 (FBAT p = 1.8e-5, EMMAX p = 6.5e-6) in the intron of the metabotropic glutamate receptor 7 (*GRM7*) gene on chromosome 3 (Figure S6 top) and rs13207753 (FBAT p = 3.4e-5, EMMAX p = 4.23e-5) in the intergenic region between Tubulin-tyrosine ligase-like protein 2 (*TTLL2*) and t-complex 10 homolog (*TCP10*) on chromosome 6 (Figure S6 bottom). The association signal for either SNP did not replicate in a previously published case-control study of 2,836 BP and 2,744 controls (GAIN [35]). However, it is noteworthy that there was a suggestive association signal located at rs58674863 (p = 3.3e-4) within the same intron of *GRM7*, 22.3 Kb distal from rs13207753 (Figure S7).

Recent large-scale genome-wide association studies have implicated a number of loci with susceptibility to bipolar disorder and schizophrenia [8], [36] and we interrogated each of these regions in the Amish pedigree. Thus, we retrieved 38 SNP association hits from a recent large-scale GWAS on BP [8] (Table S2 therein) and 24 candidate regions implicated in schizophrenia [36] (Table 3 therein). To convert the single SNP associations into candidate regions we applied an offset of 50 kb up- and downstream of each SNP. We identified 7,571 SNPs within 62 distinct candidate regions. To address the question of whether variation in these candidate regions contributes to BP susceptibility, we performed family-based burden tests using the rareFBAT test [37]. The rareFBAT test allows combined testing of the additive effects of defined sets of variants, in this case the variants within the 62 candidate regions. No significant nor suggestive (P<5×10^−4^) association was observed (Table S4), implying that these previously reported regions do not account for a major portion of the genetic risk in this pedigree. Nevertheless, the whole-genome sequencing identified a number of rare and putative damaging variants in *ANK3*, *ODZ2*, *ODZ4* and *ITIH3* genes, located within or surrounding significant genome-wide association hits for bipolar disorder (Table S5).

**Table 3:** Putative damaging exonic variants found in the five linkage regions.

Chr.	Position	Ref	Alt	dbSNP 138	Gene	GERP	1000G freq.	EVS freq.
chr2	3392295	A	G	rs11686212	*TTC15*	5.07	0.33	0.39
chr2	1168781	C	A	rs28505970	*SNTG2*	4.58	0.15	0.2
**chr4**	**1018891**	**G**	**T**	**rs4647931**	***FGFRL1***	**3.54**	**0.0058**	**0.019**
chr4	3137674	G	A	rs363075	*HTT*	4.84	0.052	0.045
**chr4**	**3446079**	**G**	**T**	**rs41264743**	***HGFAC***	**2.89**	**0.0118**	**0.0099**
chr4	5731074	C	T	rs16837598	*EVC*	4.92	0	0.063
chr4	2210065	C	A	rs2353552	*POLN*	−0.11	0.14	0.093
chr4	967191	G	A	rs17855876	*DGKQ*	1.96	0.18	.
**chr4**	**265547**	**A**	**G**	**rs150738695**	***ZNF732***	**0.977**	**0.0235**	**0.011**
chr4	1087487	G	A	rs60035268	*RNF212*	1.03	0.11	.
chr7	100365613	G	T	rs10953303	*ZAN*	3.84	0.26	0.198
chr7	100377373	G	A	rs482308	*ZAN*	4.08	0.36	0.286
**chr7**	**99995536**	**G**	**C**	**rs201649203**	***PILRA***	**3.36**	**.**	**0.00038**
**chr7**	**107569962**	**A**	**G**	**rs35915664**	***LAMB1***	**5.42**	**0.0176**	**0.01469**
**chr7**	**92763720**	**G**	**A**	**rs151304501**	***SAMD9L***	**4.59**	**0.0176**	**0.01622**
**chr7**	**107600211**	**G**	**C**	**rs80095409**	***LAMB1***	**5.31**	**0.01176**	**0.01069**
**chr7**	**92825188**	**C**	**T**	**rs145244580**	***HEPACAM2***	**4.89**	**0**	**0.00299**
**chr7**	**94917894**	**G**	**A**	**rs149869201**	***PPP1R9A***	**5.61**	**0**	**0.001**
**chr7**	**120911438**	**G**	**A**	**rs138155176**	***C7orf58***	**5.93**	**0.00588**	**0.00169**
chr7	104747899	G	T	rs117986340	*MLL5*	6.03	0.0529	0.033
chr7	92733766	C	A	rs10279499	*SAMD9*	3.37	0.0705	0.112
chr7	100389590	C	T	rs76325149	*ZAN*	2.23	0.0764	0.051
**chr7**	**87020979**	**C**	**G**	**rs111402688**	***CROT***	**5.62**	**.**	**.**
**chr7**	**100382373**	**C**	**T**	**Novel**	***ZAN***	**−0.271**	**.**	**.**
chr7	99817585	G	T	rs7786505	*PVRIG*	−3.09	0.24	0.223
chr7	106509331	C	A	rs17847825	*PIK3CG*	5.51	0.12	0.079
**chr7**	**100486464**	**T**	**G**	**rs141166290**	***UFSP1***	**3.25**	**0**	**0.00123**
**chr7**	**100371473**	**C**	**T**	**rs314299**	***ZAN***	**2.64**	**.**	**.**
chr7	100374087	A	G	rs314300	*ZAN*	4.82	0.53	0.42
chr7	89938680	C	T	rs1134956	*C7orf63*	5.51	0.53	0.50
chr16	12009304	C	A	rs11544193	*GSPT1*	4.57	0.52	0.45
**chr16**	**23634293**	**C**	**T**	**rs45551636**	***PALB2***	**5.84**	**0.0176**	**0.0178**
**chr16**	**20693663**	**C**	**G**	**rs61740631**	***ACSM1***	**3.96**	**0.02345**	**0.0161**
chr16	14029033	G	A	rs1800067	*ERCC4*	5.77	0.064	0.055
**chr16**	**11214529**	**G**	**A**	**rs200931583**	***CLEC16A***	**5.58**	**.**	**.**
**chr16**	**20352618**	**C**	**A**	**rs55772253**	***UMOD***	**?**	**0.0176**	**0.0173**
**chr16**	**21245101**	**G**	**C**	**rs143428829**	***ANKS4B***	**5.22**	**0.0058**	**0.0052**
**chr16**	**20477021**	**G**	**A**	**rs146045291**	***ACSM2A***	**1.48**	**0.018**	**0.0065**
**chr16**	**22142958**	**C**	**T**	**rs369110616**	***VWA3A***	**4.24**	**.**	**0.00016**
chr16	19548116	G	A	rs7190666	*CP110*	4.02	0.13	0.16
chr16	21051209	G	C	rs330150	*DNAH3*	2.27	0.11	0.13
chr16	19127347	C	T	rs11074362	*ITPRIPL2*	3.41	0.029	0.0596
**chr16**	**14346333**	**C**	**T**	**rs370175066**	***MKL2***	**5.84**	**.**	**7.70E-05**
chr16	23079501	G	A	rs35254998	*USP31*	5.8	0.076	0.05
chr18	10759858	T	C	Novel	*FAM38B*	5	.	.
**chr18**	**12277150**	**G**	**A**	**rs115278913**	***CIDEA***	**3.99**	**0.00588**	**0.0031**
chr18	21424991	C	A	rs17202961	*LAMA3*	5.46	0.0706	0.048

Functional impact of variants was predicted by a consensus of Polyphen2 and SIFT. Rare variants (<5% allele frequency) are shown in bold.

### Integrative linkage and association analysis

**Figure 2:**
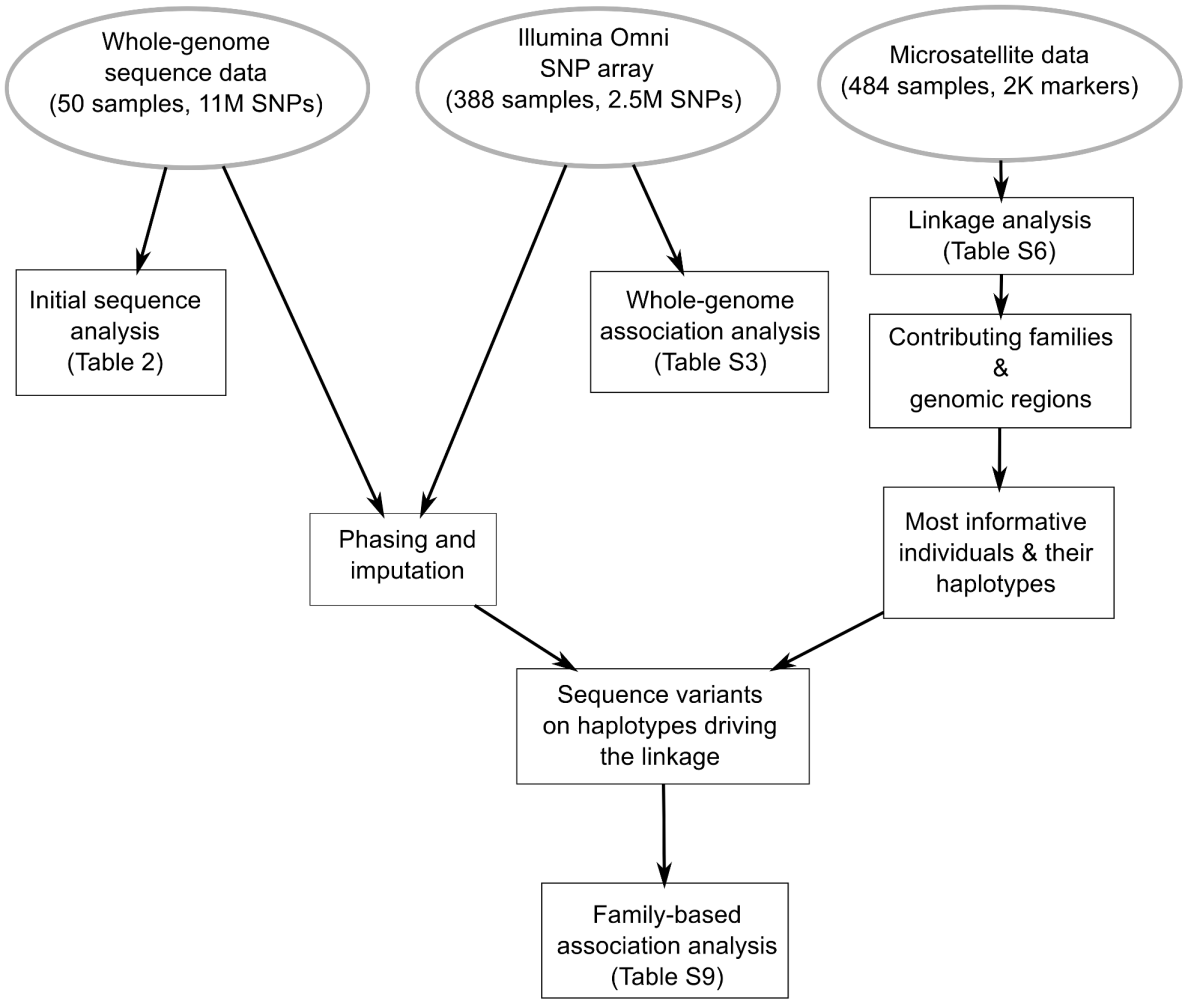
Comprehensive genetic analysis of bipolar disorder in a genetic isolate. Pipeline for the integrative analysis of microsatellite, SNP genotype and whole-genome sequence data. Linkage analysis of the ∼2K microsatellite markers is performed to identify genomic regions with evidence for linkage and to identify the nuclear families and specific haplotypes responsible for each linkage signal. Variants segregating on these haplotypes were then identified by phasing and imputation of variants found by whole-genome sequencing. In the final step, family-based association analysis is performed specifically on these variants.

To understand the role of rare variants in susceptibility to affective disorders in this pedigree, we combined linkage, association and whole genome sequence analysis (Figure 2). As expected from the results of previous studies, our results thus far suggested a polygenic mode of inheritance of BP within the Amish pedigree. In contrast to association, linkage analysis aggregates the effects of multiple, rare causal variants if they are in genomic proximity. Thus we performed linkage analysis to detect chromosomal regions that are likely to harbor susceptibility variants. The linkage analysis identified candidate genomic regions and also the subset of nuclear families supporting each linkage signal (from this point referred to as “linked families”). Further analysis of these families identified the haplotypes with maximum allele sharing in affected siblings [38]. Based on phasing and imputation of variants identified in the WGS, we then characterized each haplotype in terms of sequence variants present. As a final step, we examined association signals (from GWAS data generated using FBAT and EMMAX, see above) and carried-out family-based burden association analysis (using the rareFBAT test) on genes under the linkage peaks.

We performed linkage analysis of the 1991 microsatellite markers for 364 individuals in the extended pedigree and 7 sub-pedigrees with nuclear families with high quality imputation (see Methods). Parametric and non-parametric linkage analyses of the microsatellite genotypes were performed using the MERLIN software [39]. The parametric linkage analysis, using the multipoint LOD and heterogeneity LOD scores (HLOD) [40], [41] was performed for dominant and recessive models. Standard linear and exponential LOD scores were used for non-parametric linkage (NPL) analysis. NPL is based on allele sharing, i.e. identity-by-descent between affected siblings at a given chromosome position. Highly comparable linkage results were obtained with microsatellite genotypes and 10K SNP genotypes, with slightly higher LOD score values in microsatellite data due to the larger sample size (484 versus 388 genotyped samples).

**Figure 3:**
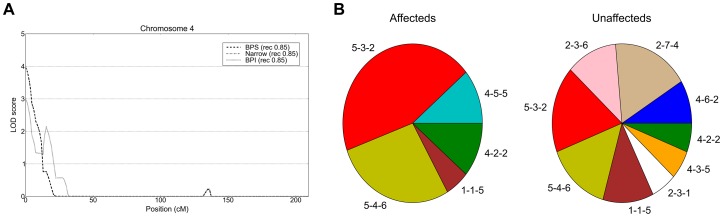
Linkage peak and haplotype analysis at the 4p16.3 locus. A) Parametric linkage analysis of chromosome 4 under a recessive model with penetrance parameter 0.85. B) Haplotype frequency distribution in affected and unaffected individuals within the three linked families at 4p16.3.

**Figure 4:**
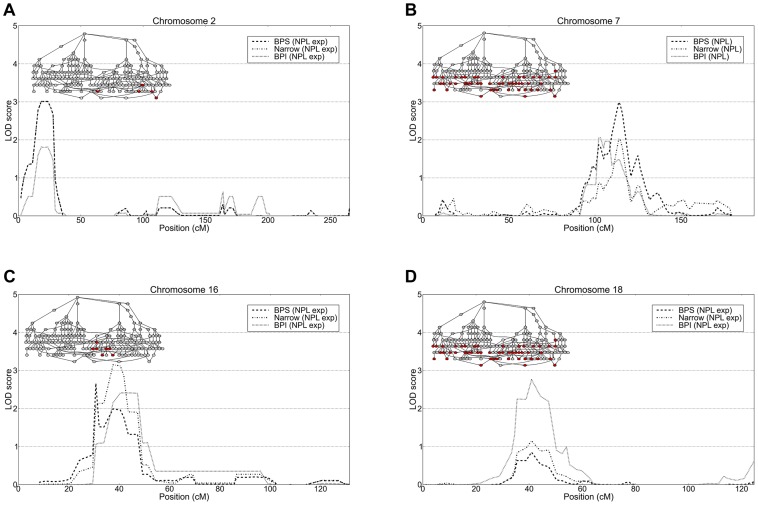
Top linkage results from non-parametric and parametric linkage analysis. Top linkage LOD scores for the three diagnostic schemes BPI, Narrow and BPS from the analysis of the extended pedigree and defined subpedigrees. Suggestive linkage (LOD>2.5) was observed for regions on A) 2p25.3-p25.1, B) 7q21.11-q31.33, C) 16p13.13-13.12 and D) 18p11.22-q12.1. The subset of nuclear families used in the analysis for each peak is shown in red in the nuclear family graph insets. Linkage on chromosomes 7 and 18 were observed using all 49 nuclear families, the peaks on chromosomes 2 and 16 were observed in subpedigrees NB4 and NB6 respectively.

Linkage analysis of all 49 nuclear families and the sub-pedigrees detected suggestive linkage (LOD>2.2) on six and eleven chromosomes respectively (Tables S6A and S6B). Within the full set of 49 nuclear families the highest linkage we observed was on 7q21 (LOD 2.99 at *D7S518*, BPS phenotype) and 18p11 (expLOD 2.76 at *D18S453*, BPI phenotype). The analysis of subpedigrees yielded nominally significant linkage peaks on 2p25 (expLOD 3.01 at *D2S2211*, Narrow phenotype) and 4p16.3 (HLOD 3.95 at *D4S3360*, Narrow phenotype) observed within subpedigree 310/410 as well as 16p13 (expLOD 3.15 at *D16S3127*) within sub-pedigree 310 (Figures 3A and 4). Several of the detected peaks were reported in previous linkage studies of bipolar disorder. The 7q21 region showed significant evidence for linkage (P = 0.0003) in a joint meta-analysis of data from 11 BP and 18 schizophrenia studies [42]. Interestingly, the authors note that the signal at the locus “appears to be due to the inclusion of results for the broad susceptibility models such as those including unipolar depression”. This is consistent with the strongest signal being observed under the BPS phenotype in our data. Moreover, the 7q21.3-q22.3 region has been reported to yield suggestive linkage (NPL LOD = 2.28) for a data set of Ashkenazi Jews and Belgian Europeans [43]. The 18p11.22 region is one among the most frequently reported bipolar disorder linkage peaks. Linkage in the 18p11 region was first reported based on affected sibling analysis of 22 BP families [44] and subsequently confirmed by a study of 28 families with apparent unilineal transmission of BP [45]. Also, a study of the effect of age-of-onset by ordered subset analysis found significant linkage at the 18p11.2 locus [46]. In addition to these findings, suggestive linkage was reported in a non-parametric linkage analysis of a data set of 22 Caucasian families [47] and in an association study of 363 parent-child trios of Caucasian descent [48]. Finally, the microsatellite marker with the highest linkage signal on chromosome 18 detected in our study (*D18S453*) maps less than 1 Mb distal to the translocation breakpoint [t(18;21)(p11.1;p11.1)] segregating with schizophrenia in a seven member family [49]. A number of groups reported evidence for linkage for bipolar and other psychiatric disorders in the 4p15.2–4p16.3 region (see references in [47], [50]–[53]), including a genome-wide linkage study of mental health wellness in the Old Order Amish which found significant evidence for linkage on chromosome 4p, about 5 Mb distal of the peak we detected [53].

Importantly, the analysis of identity-by-state sharing under each linkage peak identified considerable haplotype heterogeneity, with multiple contributing haplotypes across the linked nuclear families at each peak (Table S7). The notable exception to this was the three-marker haplotype on 4p16.3 (*D4S3360-D4S2936-D4S412*) covering the highest observed linkage peak (HLOD 3.96). Here among the four contributing haplotypes (4-5-5, 5-4-6, 5-3-2, 4-2-2 at above listed microsatellite loci), a single haplotype, 5-3-2, was shared by 3 out of 4 linked nuclear families. The 5-3-2 haplotype was present in 9/10 affected individuals within the contributing families. There was significant haplotype association of the 5-3-2 haplotype (FBAT p = 0.0267) as well as for the global omnibus test (FBAT global p = 0.0291) in affected individuals within linked families (Table S8, Figure 3B).

Whole-genome sequencing identified multiple non-synonymous variants under each peak (Table 3). Many of these exonic, putative damaging variants (by consensus of Polyphen2 and SIFT) are common (MAF>5%) in European populations based on 1000 Genomes and the EVS data. Such variants are found at 2p25 (in the *SNTG2* and *TTC15* genes) and 4p16.3 (in *POLN* and *EVC* genes). However a significant number of variants under the linkage peaks are either novel or rare (e.g. *LAMB1*, *MKL2*, *ZNF732*, *ZAN* and *ANKS4B*) or moderately frequent (e.g *ERCC4* and *MLL5*). Using the current level of resolution, it is difficult to evaluate whether these variants contribute to nominal association signals within the five regions detected by the analysis of SNP array data using EMMAX and FBAT (Figure S8).

We also analyzed sequence variation on linkage-driving haplotypes by focusing on exonic missense variants and SNPs associated with the expression level of genes under linkage peaks (‘eSNPs’ see Methods) [54]. To test for association in the presence of allelic heterogeneity we then performed rareFBAT burden tests for all genes under the five linkage peaks with either exonic missense or regulatory eSNP variants (Figure S8). While the included variants were selected based on their presence on linkage-driving haplotypes, the tests were performed on data from the entire pedigree, i.e. both linked and unlinked families. The standard Null hypothesis of the rareFBAT test is “no linkage and no association”. Since there was a subset of linked families at each linkage peak, this Null might lead to slightly liberal p-values. To account for this we also report p-values for testing the Null hypothesis of “linkage and no association”, which can be seen as a conservative correction as the majority of families are unlinked (see Methods). Forty-two (42) of the 543 genes under the five linkage peaks were nominally associated (P< = 0.05; Figure S8; Tables S9, S10, S11, S12, S13, S14). However, significance was not retained after correction for multiple testing by False Discovery Rate (FDR) [55]. We performed Ingenuity Pathway Analysis (IPA) of nominally significant genes but observed no strong enrichment for a specific pathway, biological function or tissue-specific gene expression (see Figure S9 for an example IPA analysis of the top 100 genes on Table S2). Under the 7q21 peak, the most strongly associated genes were the tectonin beta-propeller repeat containing 1 (*TECPR1*, *P* = 2.8×10^−5^) and the neighboring distal-less homeobox 5 and 6 genes (*DLX5* and *DLX6*) (P = 0.008 and P = 0.003 respectively). The most strongly associated gene under the 16p13 peak was the ATP-binding cassette, sub-family C, member 6 (*ABCC6*) gene (P = 0.00055). The 2p25 region harbors the integrin beta 1 binding protein 1 (*ITGB1BP1*) gene, which is involved in cell adhesion. The class of cell adhesion molecules has been implicated to play a role in bipolar disorder in multiple gene set enrichment analyses studies [56], [57]. Thus, while our results do not allow the unequivocal identification of a causal gene under the identified linkage peaks, the gene-wise burden tests analysis yields a number of plausible candidate genes (Table S9).

## Discussion

We employed several strategies to comprehensively delineate the role of both common and rare variants as susceptibility alleles for bipolar affective disorder in a large extended pedigree with a high incidence of the disorder. The role of common variants was initially explored using family-based genome-wide association analysis of high-density SNP genotypes for the entire family. To address the role of rare variants we employed linkage analysis to prioritize sequence variants from WGS on specific haplotypes enriched in affected subjects. A combination of WGS with dense SNP genotypes that allow long-range haplotype phasing has been successfully utilized to identify variants that influence risk for sick sinus syndrome, gout, gliomas, ovarian cancer and Alzheimer's disease [58]–[62]. However, it appears this has not yet been attempted for a genetically and phenotypically complex mental disorder. Our extensive analysis of a single exceptionally large family with affective disorder revealed an unanticipated level of genetic complexity with no convergence of evidence in support for a limited number of loci conferring disease risk in this family.

Our linkage analysis identified several nominally significant peaks in different sub-pedigrees under different disease models. These results are consistent with a polygenic mode of inheritance contributing to the disease susceptibility and phenotypic presentation. Polygenic inheritance usually implies that interacting risk factors in several genes across the genome lead to disease susceptibility. Our fine mapping of imputed variants in regions with the strongest linkage and association signals did not reveal a single SNP (or haplotype) but rather several non-coding and exonic SNPs. Due to the long haplotype blocks observed in founder populations, a linkage signal may reflect a combined effect of multiple causal alleles within the same chromosomal region as commonly described in the dissection of behavioral traits in model organisms [63]–[65]. The combined high-density SNP genotyping, microsatellite genotyping, and whole-genome sequencing are expected to capture nearly all of the genetic variants co-segregating with the BPI phenotype. The lack of any single variant or haplotype exhibiting a significant linkage signal under both parametric and non-parametric analysis suggests that the BP1 disorder within this pedigree is likely to be genetically heterogeneous and caused by aggregation of different moderately frequent alleles in different sub-pedigrees.

Using WGS we identified a number of non-synonymous, likely deleterious variants that are rare in the 1000 Genomes Project dataset (2%) but present in 10–30% of the BP subjects and their first-degree relatives. Such differences in allele frequency, resulting from a population bottleneck and expansion from a relatively small number of founders, have been previously described in several isolated populations (see [66], [67] for reviews). Understanding the phenotypic consequences of these Amish-enriched variants will be important not only in the context of their potential contribution as risk factors (or modifiers) for mental illness but also for other, yet undiagnosed disorders segregating in the Amish [68]. For example, the variant rs113270504, which leads to a Val540Met substitution in the G Protein-Coupled Receptor 124 (GPR124), is rare in non-Amish populations (<2% in 1000 Genomes and EVS). The corresponding gene is essential for angiogenesis in the central nervous system and the development of the blood-brain barrier [69], [70]. Another variant, rs78247304 in *KCNH7* (Arg394His), potassium voltage-gated channel, subfamily H, member 7, is of considerable interest. Voltage-gated ion channels have previously been implicated in a number of psychiatric disorders, including bipolar disorder, due to their known involvement in neuronal excitability and synaptic transmission [71]. Association at the *KCNH7* locus with bipolar disorder was also reported at rs6736615, an intronic variant, in a recent case-control study of 400 Taiwanese subjects [72]. Moreover, an exome-sequencing study of a small number of Amish subjects with mental illness identified rs78247304 as a likely risk variant and showed that the Arg394His substitution leads to altered voltage dependence and activation kinetics of the channel in patch clamp experiments [73]. In addition, we have identified three genes (*ADAM18, INSL6, IFT81*) that play roles in spermatogenesis and fertilization. To determine whether mutations in these genes may underlie bipolar disorder and/or other complex traits, it will be necessary to explore additional Amish families with mental illness and comorbid medical conditions.

Our analysis of previously published genome-wide significant GWAS loci did not show any convergence with the results from our family-based GWAS. Recently, two publications employed whole exome sequencing to identify rare variants associated with bipolar disorder using a family-based design [74], [75]. The analysis of a single family with three affected daughters and an unaffected brother identified variants that segregate with bipolar disorder (and are not present in 200 controls) in eight brain expressed genes [74]. We examined genetic variation in these genes in our data and identified several low frequency, potentially damaging variants in *JMJD1C*, also known as thyroid hormone receptor beta-biding protein8 (*TRIP8*), involved in hormone-dependant transcriptional regulation. Among several genes identified in the second publication [75], the most striking are variants in Odd oz 2, *ODZ2* (also known as *TEN-M2*). A rare variant in this gene and in a paralogous gene *ODZ4*, with an intronic SNP (rs12576775) associated with bipolar disorder at a genome-wide level [8], segregate in this large Amish pedigree, but again do not reside in regions with the nominal association or linkage signal (Table S5).

Isolated founder populations provide a unique opportunity to evaluate the genetic architecture of human disease [67]. Family-based linkage scans provide initial insights into the number of disease conferring loci, while association studies can define the role of common alleles or alleles that are rare in other populations, but moderately frequent in the isolate. The lack of statistical power observed in our study may be due to the modest number of family members and (or) a small effect size of risk-alleles. Therefore, to establish a causal link between specific variants and disease risk will require more in-depth analysis in additional families and population-based cohorts in the same or related Amish communities. Moreover, to address the high level of genetic heterogeneity, it will be necessary to phenotypically dissect the clinical diagnoses and explore subtle differences in manifestation or medical comorbidities between subpedigrees [76]. The Iceland-based deCODE study highlights the value of combining genomic characterization of a genetic isolate with the comprehensive phenotypic data to enable identification of variants associated with several human diseases and other traits [58], [77], [78]. Recently a systematic framework for studying the genetic architecture of complex traits has been proposed [79]. By simulating data under a range of disease models, the method determines which genetic architectures are consistent with the empirical data. For example, the study shows that while the number of known risk loci for type 2 diabetes is consistent with a range of disease architectures, disease models with extremely low or high values for the true number of risk loci can be ruled out. Performing a similar analysis of bipolar disorder would require the integration of linkage, GWAS and, crucially, several of the upcoming next-generation sequencing studies.

Leveraging family-based design with the population-based studies of BP-associated quantitative traits in a founder population will be critical for effective gene and human-genetics driven drug discovery. Although candidates detected by our comprehensive approach were not significant after the correction for multiple testing, we provide supportive evidence and a biological rational for further investigating these variants in large cohorts, especially when more genome and exome data become available.

## Methods

### Ethics statement, sample subjects and genotyping

The genetic-epidemiologic study of bipolar disorder among the Old Order Amish, in settlements throughout Pennsylvania, was documented extensively, including ascertainment protocols and diagnostic methods [17], [18], [80]. Ascertainment of patient cases used a “scribe network” with assigned codes to ensure patient confidentiality. Structured interviews (SADS-L) [81], [82] were conducted with the patient and close others. Signed, informed consents were obtained to access medical records. Two forms were used: a) one with yearly Institutional Review Board (IRB, University of Miami) approval adhering to special guidelines because the Amish are defined as a vulnerable population; and b) a second using state approved, medical record consent forms for specific mental health clinics and psychiatric hospitals throughout central Pennsylvania. These were abstracted and collated for five members of the Psychiatric Board who were “blind” to patient names, pedigree, address, admission/discharge diagnosis and treatment. Abstracted medical records and SADS-L interview materials were reviewed, often separated by several years, as a reliability check on diagnosis based on the two sources of information. The Psychiatric Board members used strict Research Diagnostic Criteria (RDC) [23], [82] and the Diagnostic and Statistical Manual of Mental Disorders, 4^th^ Edition (DSM-IV) for uniform clinical criteria [17], [24]. Clinical assessments by the Psychiatric Board have continued since 1977 and BPI patients and relatives in the genetic study followed annually. Although there is a spectrum of Major Affective Disorders in the extended Amish pedigrees, the majority of “affected” individuals are diagnosed as either BPI, BPII, or Major Depressive Disorder (MDD, recurrent) with only two subjects with Schizoaffective Disorder, BP Subtype. Course-of-illness over time is essential for ascertainment of “onsets” given a variable age of onset. Onset of illness and the value of documented “well” relatives of a BPI as controls have been previously reported [80]. The Coriell Institute of Medical Research (CIMR) in development of their national cell repository established Lymphoblastoid cell lines (http://ccr.coriell.org/Sections/Collections/NIGMS/AmishStudy.aspx?PgId=600). Collection of blood/tissue samples followed diagnostic consensus, using two informed consent forms: a) one with annual Univ. Miami IRB approval defining (with language appropriate for Old Order Amish) how their cells would be preserved for medical research on Major Affective Disorders; and, b) the Informed Consent Form required by the Institute for Medical Research (CIMR), later Coriell - National Institute for General Medical Sciences (NIGMS) Human Genetic Cell Repository (HGCR). Since completion of the Amish Study BPI cell collection at CIMR (JAE curator), the NIGMS-HGCR consent form has undergone revisions. The CIMR/NIGMS Informed Consent Forms makes no reference to specific diseases, types of genetic analysis that will be performed in future studies (type of genetic markers, candidate gene or Whole Genome Sequencing and so on). Analysis of whole-genome re-sequencing data from consented individuals in this pedigree was also approved by the IRB of the Weill Cornell Medical College and the Perelman School of Medicine at the University of Pennsylvania.

Based on genealogy the whole pedigree is subdivided into four large subpedigrees (110, 210, 310 and 410) [18], with each subpedigree comprised of multiple nuclear families. Forty-nine nuclear families were selected with at least one confirmed BPI case for linkage analysis. These families were spread across all four previously defined subpedigrees. The 364 subjects in these 49 nuclear families included: 62 BPI, 15 BPII, 15 Major Depressive Disorder, recurrent, and 220 unaffected/well subjects. Additionally, there were 52 individuals with minor mental illness (such as minor depression) treated as having an “unknown” diagnosis. The average age-of-onset for BPI patients was 24.6. In addition to the full set of 49 nuclear families, we also analyzed seven smaller clusters of closely related families. These so called “neighborhoods” (NBs) were defined by selecting families around parent-child trios with whole-genome sequence (Table S15).

### Nuclear family graph

To visualize the high-level structure of the pedigree and the degree of relatedness of nuclear families we constructed the nuclear family graph (Figure 1A). The nuclear family graph is a directed acyclic graph *G* = (*V*, *E*) where each node in *V* represents a nuclear family in the pedigree. Edges in *E* correspond to parent-child relationships connecting the nuclear families. If there is an individual who is a child in one family and the parent in another, we connect the two families by a directed edge. The nuclear family graph also protects the privacy of individuals by allowing the visualization of the relatedness between families without revealing family structure (sex, number of children).

### Whole genome sequencing

Whole genome sequencing (WGS) was performed by Complete Genomics Inc. (CGI; Mountain View, CA) using a sequence-by-ligation method [26]. Paired-end reads of length 70 bp (35 bp at each end) were mapped to the National Center for Biotechnology Information (NCBI) human reference genome (build 37.2) using a Bayesian mapping pipeline [83]. Variant calls were performed by CGI using version 2.0.3.1 of their pipeline. The overall ratio of transitions (Ti) to transversions (Tv) was ∼2.15 in each sample. Within the confines of our study we focused on SNP variants, as the reliability of the called genotypes is higher than for indels [26]. False discovery rate estimates for SNP calls of the CGI platform are 0.2–0.6% [26]. Gene annotations were based on the NCBI build 37.2 seq_gene file contained in a NCBI annotation build. The variant calls within the WGS were processed using the *cgatools* software (version 1.5.0, build 31) made available by CGI. The *listvar* tool was used to generate a master list of the 11.1M variants present in the 50 Amish samples. The *testvar* tool was used to determine presence and absence of each variant within the 50 Amish WGS. Only variants with high variant call scores (“VQHIGH” tag in the data files) were included. Variants that were observed in a single WGS were excluded.

In each sample sequencing depths was averaged within 100 Kb windows across the genome. Median sequence coverage over all windows across the 50 samples was ∼50×. Within each sample averaged sequencing depth was >40× for ∼70% of the genome and >10× ∼98% in all 50 sequenced individuals (Figure S2). Coverage for the coding region was comparable to the genome average: > = 40× for an average of 75.5% of the exome and > = 10× for 97.1%. Coverage did not show substantial degradation for different GC content (Figure S10). The ancestral allele was identified based on annotation from the 1000 Genomes Project; for novel variants the reference allele was used. Additional allele frequencies were obtained from the Exome Variant Server (EVS) database (http://evs.gs.washington.edu/EVS/). For novel variants in our Amish WGS the allele frequencies in the Complete Genomics Diversity Panel (http://www.completegenomics.com/public-data/), a set of WGS from 54 unrelated HapMap samples, was used as a technical control to guard against platform specific false positive calls. Predictions of functional impact of exonic missense variants using Polyphen2 [29] and SIFT [30] were retrieved from the dbNSFP database (version 2.0b3) [84].

### Genome-wide association analysis

Genotyping of 394 samples from the extended Amish pedigree using Illumina Omni 2.5 M SNP arrays was performed at the Center for Applied Genomics (Children's Hospital of Pennsylvania, Philadelphia, PA). We performed rigorous quality control of the raw genotype calls by applying a series of filters on both markers and samples using PLINK (http://pngu.mgh.harvard.edu/~purcell/plink/). The initial dataset contained 2,379,855 SNPs and 394 samples. The following filters were applied in sequence; the numbers of markers or samples excluded is given in parentheses: a) exclude SNPs with missing rate >0.5 (19,435), b) exclude samples with missing rate >0.02 (6), c) exclude SNPs with missing rate >0.02 (31,678), d) exclude SNPs with MAF<0.02 (1,018,805), e) exclude SNPs with informative missingness p<1e-6 (0), f) exclude SNPs with Hardy-Weinberg equilibrium p<1-e6 (0), g) exclude individuals with >5% Mendel errors (0) and h) exclude SNPs with >1% Mendel errors (1334). After quality control we retained 1,309,937 SNPs and 388 samples. A single sample with WGS was removed by quality control filtering. For the remaining 49 samples with both WGS and SNP array genotypes, we evaluated concordance using the cgatools snpdiff program (http://cgatools.sourceforge.net/). We found an average concordance of 0.996 (SD 9.1e-05) in the called variants, suggesting a very high consistency of the WGS and SNP array datasets.

CNVs were called by PennCNV [25], using the GC model wave adjustment [85]. CNVs were removed if they had a value >0.30 standard deviation of LRR (LRRSD), a waviness factor (WF) value >0.05, or <5 SNPs. Samples that had a total CNV number greater than 3 SD from the mean, or samples that showed evidence of aneuploidy, were also excluded.

We performed genome-wide association analysis using two different methods: (a) EMMAX [32] (version from March 7th 2010), a method for case-control analysis with correction for relatedness among samples using mixed models and (b) FBAT [33] (version v2.0.4Q), an extension of the classical transmission distortion test (TDT) [34] to larger families. Imputation of GAIN data were conducted on 2,191 cases and 1,434 controls previously genotyped as part of [86] using Impute 2.1 [87] based the 1000 Genomes June 2011 Haplotype interim release containing 37M SNPs using Ne parameter of 20,000, a genotype threshold of 0.9, and followed by calculation of allelic association using PLINK 1.07.

### Analysis of kinship coefficients

We calculated pair-wise kinship coefficients based on the SNP array genotypes using the KING software [28]. As described in the KING paper, expected ranges of kinship coefficients are >0.354 for duplicate samples/monozygotic twins, [0.177–0.354] for 1^st^ degree relatives, [0.0884–0.177] for 2^nd^ degree relative, [0.0442–0.0884] for 3^rd^ degree relatives and <0.0442 for unrelated subjects. Observed ranges of kinship coefficients for known family relationships in the extended pedigree match well to these expectations (Figure 1C). As an outside control and reference for unrelated subjects, we also obtained kinship coefficients for 569 parent pairs using Illumina 550 k SNP genotypes from the Autism Genetics Research Exchange (AGRE) collection [88]. To address the presence of hidden relatedness, we compared kinship coefficient estimates based on SNP genotypes with the theoretical kinship derived solely from pedigree information. We observe a slight overall excess of kinship coefficients, signifying a degree of hidden relatedness not accounted for in the available pedigree information, i.e. the mean of pair-wise kinship coefficient estimates shows average kinship coefficients of 0.015 for an expected value of 0.01. To estimate the overall level of inbreeding and identify consanguineous nuclear families we calculated the relatedness between 32 pairs of parents where genotype data was available for both parents.

### Phasing and imputation

We performed phasing and imputation of variants identified by WGS into the Omni 2.5 M SNP genotypes using the Genotype Imputation Given Inheritance (GIGI) software [89] version 1.02. GIGI performs imputation of dense genotypes in large pedigrees based on a sparse panel of framework markers using a Markov Chain Monte Carlo approach. Importantly, since GIGI does not make use of a reference panel we avoid introducing potential biases due to genetic differences in the Amish and, for instance, a European reference population. The genetic map in Haldane centiMorgan (cM) coordinates for the Omni 2.5 M array was retrieved from the Rutgers Combined Linkage-Physical map (http://compgen.rutgers.edu/maps) [90]. Framework markers were chosen evenly spaced 0.3 cM among array SNPs without Mendelian errors and >0.25 minor allele frequency. The first step in the imputation consists of sampling of 5000 inheritance vectors (IV) for the framework markers using the MORGAN software. To make this feasible for the large OOA pedigree, imputation within the identified linkage regions was performed separately for the six sub pedigrees (110C, 210, 110L, 410, 110R, 310). The informativeness of framework markers with respect to recombination events was quantified using the MERLIN software. The mean information content over all framework markers was 0.987 (SD 0.0092), suggesting that the selected frameworks were highly informative. To assess the quality of the imputed genotypes we applied a similar setup to the GIGI paper [89]: variants with SNP array genotypes that were not part of the framework were blocked for all samples except those with whole genome sequence available and subsequently imputed. Imputation performance can then be evaluated by the concordance of imputed and SNP array genotype calls, as well as the call rate of imputed genotypes for different thresholds on the genotype imputation posterior probability. We evaluated performance for all samples (n = 388) (Figure S11A), individuals in families in the neighborhood of WGS subjects (i.e. on step removed in the nuclear family graph) (n = 268) (Figure S11B) and siblings, parents or children of individuals with WGS present (n = 171) (Figures S11C). Overall performance is comparable to the published report [89]. For a threshold on the genotype imputation posterior probability of 0.85, we observed overall concordance of ∼0.96 with a call rate of ∼0.50 (Figure S11A). As expected, imputation performance increases for sub-pedigrees with a higher number of samples with WGS. At the same threshold of 0.85, families in the neighborhood of families with WGS show concordance of ∼0.97 with call rate ∼0.65 (Figure S11B) and when considering only nuclear families with WGS samples this further improves to concordance ∼0.99 and call rate ∼0.87 (Figure S11C). Based on these results we chose a threshold of 0.85 for calling imputed genotypes.

### Linkage analysis

Parametric and non-parametric linkage analyses were performed using the MERLIN software version 1.1.2 [39]. MERLIN provides exact identity-by-descent (IBD) solutions based on the Lander-Green algorithm, which allows for the simultaneous analysis of the entire marker panel but is limited to pedigrees of small to medium size. Thus the Amish pedigree was split into nuclear families as the basic unit of analysis. In the parametric case, linkage analysis was performed using the multipoint HLOD score setting, which allows for the presence of unlinked nuclear families. A panel of 2K microsatellite markers for linkage analysis was genotyped at deCODE Genetics (Reykjavik, Iceland). The average information content per marker assessed with the entropy measure in MERLIN was 0.89 (SD 0.022) indicating that the marker panel was highly informative. We employed the LD modeling option in MERLIN although this did not greatly affect the linkage results. As part of quality control we employed the MERLIN error correction to block unlikely genotype calls. For the parametric linkage analysis we applied dominant and recessive models. The parameterization for zero, one and two copies of the disease alleles was (0.0001, 0.0001, 0.85) for the recessive model and (0.0001, 0.85, 0.85) for the dominant model. The disease allele frequency parameter was set to 0.01. For non-parametric linkage the NPL-Pairs scoring function was used with both the linear and exponential model. The linear model is most suited for the detection of small increases of allele sharing in a large number of families. The exponential model is more powerful in the situation of substantial increases in allele sharing within a small number of families. Study-specific critical values for the non-parametric linkage were determined automatically by applying autoregressive models to the correlation between standard normal statistics at adjacent map points [91]. A threshold for suggestive linkage of LOD = 2.2 was selected. In addition to the analysis of all nuclear families in the pedigree, we also analyzed the selected neighborhoods (NB1-NB7) (Table S15). As the neighborhoods were centered on families with WGS available, we obtained small, closely related groups of nuclear families with high-quality genotype imputation (Figure S11B).

### Gene burden tests

To test for gene-wise burden under an additive model we applied a family-based test of additive burden (rareFBAT) [37] implemented in the FBAT software version v2.0.4Q. The contribution of variants to the overall test score was weighted inversely to their allele frequency, i.e. rare variants contributed more (-v1 option). The default Null hypothesis of FBAT is “no linkage and no association”. In the presence of linkage FBAT can empirically correct the estimates for the variance-covariance structure (-e option) to test the Null of “linkage and no association”.

### Heritability of bipolar disorders

The heritability of bipolar disorder was estimated using the Sequential Oligogenic Linkage Analysis Routines (SOLAR) [27] software version 6.6.2. The SOLAR “polygenic” command performs a variance components analysis to determine the fraction of phenotypic variance accounted for by additive genetic effects (i.e. narrow sense heritability or h^2^). Age and sex were used as covariates.

### Expression quantitative trait locus QTL dataset

Sequence variants that significantly impact gene expression in the human brain ([92], GEO accession: GSE8919) were retrieved from data in a recent re-analysis of microarray data from 11 eQTL studies over 7 different tissues [54]. Here, we used a fairly inclusive definition of regulatory activity by including all SNPs showing positive evidence for eSNP activity. To extend the eSNP annotations to novel and rare variants found in the whole genome sequence data, variants within 50 bp of known eSNPs were also included as potential regulatory variants.

### Supplemental data

Supplemental Data include eleven figures and fifteen tables.

## Supporting Information

Figure S1 Family relationships among 50 subjects with whole-genome sequence.
(JPG)

Figure S2 Weighted sequence coverage for the whole genome (blue) and exome (red) of WGS of 50 Amish samples.
(PNG)

Figure S3 Pairwise kinship coefficient matrices for 388 samples with Omni2.5 SNP genotypes. A) Kinship coefficients based on known pedigree relationships. B) Estimated kinships coefficients based on 1.3M SNP genotypes.
(TIF)

Figure S4 Quantile-Quantile plots of genome-wide association analysis. A) FBAT analysis and B) EMMAX analysis.
(TIF)

Figure S5 Manhattan plots of genome-wide association analysis of 388 samples from the extended Amish pedigree. A) Family-based association analysis using FBAT, B) Mixed model case-control analysis with correction for relatedness using EMMAX.
(TIF)

Figure S6 FBAT (left) and EMMAX (right) association results at the *GRM7* (top) and *TTLL2-TCP10* loci (bottom) (plots generated with LocusZoom).
(TIF)

Figure S7 LocusZoom plots of case-control association for 2,836 BP cases and 2,744 controls (GAIN) at the *GRM7* (left) and *TTLL2-TCP10* (right) loci.
(PNG)

Figure S8 SNP-based and gene-wise burden association tests in the linkage regions. For each of the five identified linkage regions (A) 2p25, B) 4p16.3, C) 7q21, D) 16p13 and E) 18p11) association results with EMMAX and FBAT using Omni2.5 SNP array data (top and middle panel) are shown. The bottom panels display the results of gene-wise burden tests of exonic and regulatory variants using the rareFBAT test based on imputed WGS and Omni2.5 genotype data. The x-axis shows the chromosomal position in Mb, the y-axis the -log(P-values) for the respective method. Each data point corresponds to a single SNP in the EMMAX and FBAT panels and a combined burden test for exonic and regulatory variants in a single gene in the rare FBAT panels.
(TIF)

Figure S9 Example of an Ingenuity Pathway Analysis (IPA) of the top 100 genes from table S1. No significant enrichment for a specific pathway, biological function or tissue-specific gene expression was observed.
(TIF)

Figure S10 Normalized coverage (i.e. coverage relative to the genome average) for different GC content percentiles. GC content was calculated for bins of 501 bp across the genome. It can be seen that the normalized coverage only drops off at the very extremes of the GC content distribution in all 50 samples.
(PNG)

Figure S11 Evaluation of imputation performance for different thresholds on the genotype posterior probability. Concordance between imputed and SNP array genotypes (red) and call rate of the imputed genotypes (blue) are shown. The plot shows averages over A) all samples in the dataset, B) families in the neighborhood of WGS samples and C) first degree relatives of subjects with WGS.
(TIF)

Table S1 Top 50 most conserved rare exonic variants by GERP conservation score.
(XLSX)

Table S2 514 Amish-specific putative damaging exonic missense variants. Exonic missense variants, identified in 50 subjects with WGS, were filtered by <2% allele frequency in 1000 Genomes, EVS and 54 HapMap WGS. Functional impact was assessed by consensus of Polyphen2 and SIFT.
(XLSX)

Table S3 Top SNPs from genome-wide association analysis with FBAT and EMMAX association analysis of 1.3M SNPs for 388 subjects from the extended pedigree. Only SNPs with p< = 5e-4 are shown.
(DOCX)

Table S4 Result from rareFBAT burden test analysis of 62 candidate regions for BP and schizophrenia identified by recent GWAS studies. The table shows chromosomal region, number of Omin2.5 SNP included, rareFBAT P-value and (if applicable) the previously reported associated SNP.
(XLS)

Table S5 Exonic missense variants in genome-wide significant BP association hits in the *ANK3*, *ODZ4* and *NEK4-ITIH1-ITIH3-ITIH4* region as well as 24 genes reported in two recent whole-exome sequencing studies.
(XLS)

Table S6 A) Linkage results from the analysis of 49 nuclear families from the extended pedigree and B) linkage results from the analysis of defined subpedigrees. The table lists the chromosomal region, top LOD marker, length of peak region for a 2 LOD confidence interval, phenotype under which maximum linkage was observed, linear non-parametric LOD score (NPL LOD), exponential non-parametric LOD score (NPL expLOD), parametric LOD score, parametric HLOD score and the alpha value denoting the fraction of linked families in the HLOD computations.
(DOC)

Table S7 Number of linked families and contributing haplotypes for five identified linkage regions.
(DOC)

Table S8 FBAT haplotype (*D4S3360, D4S2936, D4S412*) association test results for linked families in the 4p16 linkage region under an additive model. Haplotypes identified by allele sharing in affected siblings are shown in bold. Significant haplotype association (p = 0.0267) is observed for the 5-3-2 haplotype. The global FBAT haplotype association test also was significant with p = 0.0291.
(DOC)

Table S9 Genes within the five linkage regions with nominally significant burden test association (P<0.05). The table shows the peak region, Gene, number of exonic and regulatory SNPs included in the burden test and rareFBAT P-values for the Null of hypotheses of “No linkage, no association” and “linkage and no association”.
(DOCX)

Table S10 Result from rareFBAT burden test analysis of 47 genes under the 2p25 linkage peak. Exonic missense and eSNP variants were included in the test for each gene.
(XLS)

Table S11 Result from rareFBAT burden test analysis of 83 genes under the 4p16.3 linkage peak. Exonic missense and eSNP variants were included in the test for each gene.
(XLS)

Table S12 Result from rareFBAT burden test analysis of 249 genes under the 7q21 linkage peak. Exonic missense and eSNP variants were included in the test for each gene.
(XLS)

Table S13 Result from rareFBAT burden test analysis of 98 genes under the 16p13 linkage peak. Exonic missense and eSNP variants were included in the test for each gene.
(XLS)

Table S14 Result from rareFBAT burden test analysis of 66 genes under the 18p11 linkage peak. Exonic missense and eSNP variants were included in the test for each gene.
(XLS)

Table S15 Numbers of families and subjects with WGS data for the entire pedigree and seven more homogeneous subpedigrees chosen based on family relationship and imputation quality.
(DOC)
