# Supplementary figures and images for: Genomic View of Bipolar Disorder Revealed by Whole Genome Sequencing in a Genetic Isolate
Source: PLoS Genet. 2014 Mar 13;10(3):e1004229. doi: 10.1371/journal.pgen.1004229 (PMC3953017; doi:10.1371/journal.pgen.1004229)

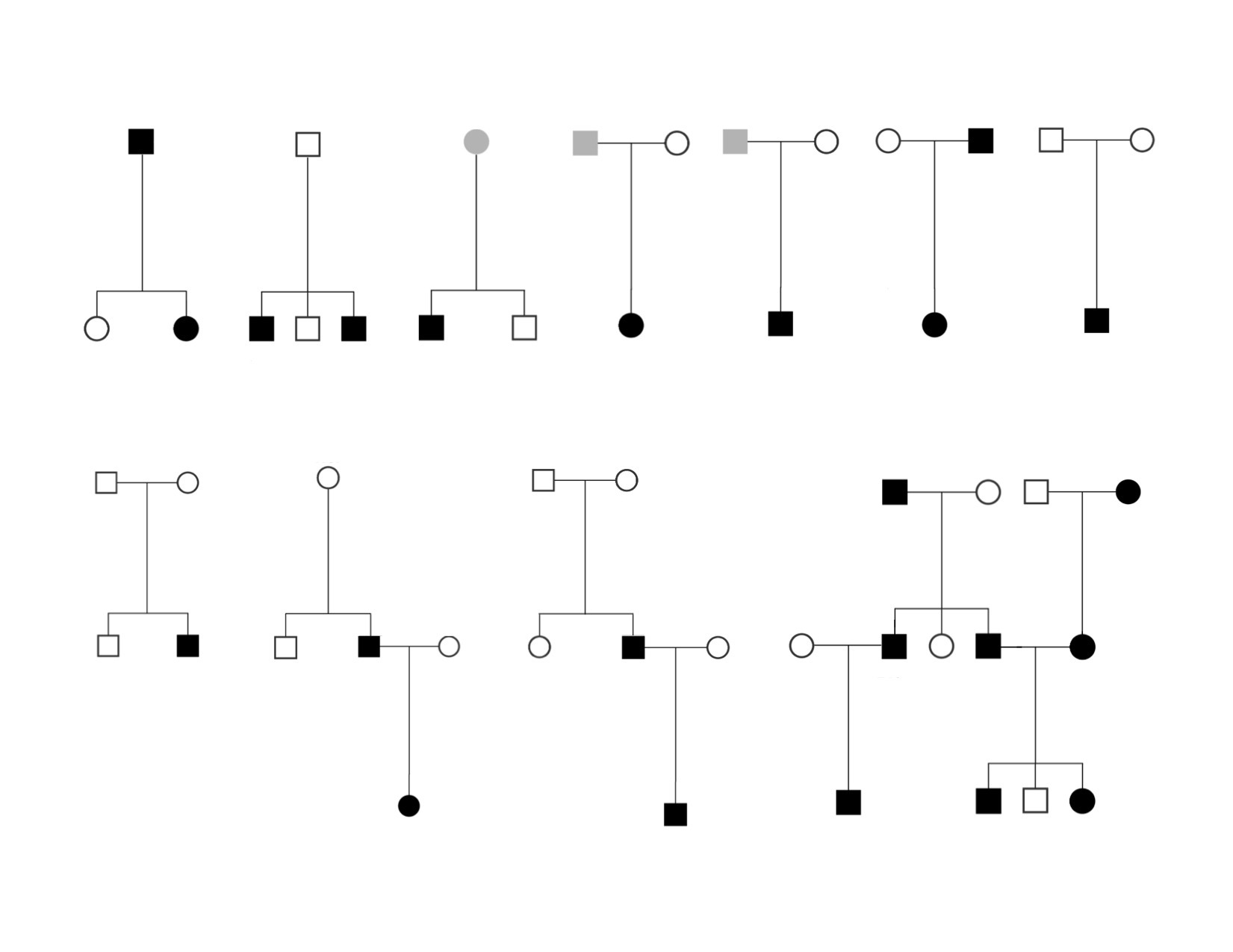

Supplement: Figure S1 — Family relationships among 50 subjects with whole-genome sequence. (JPG) [file pgen.1004229.s001.jpg]

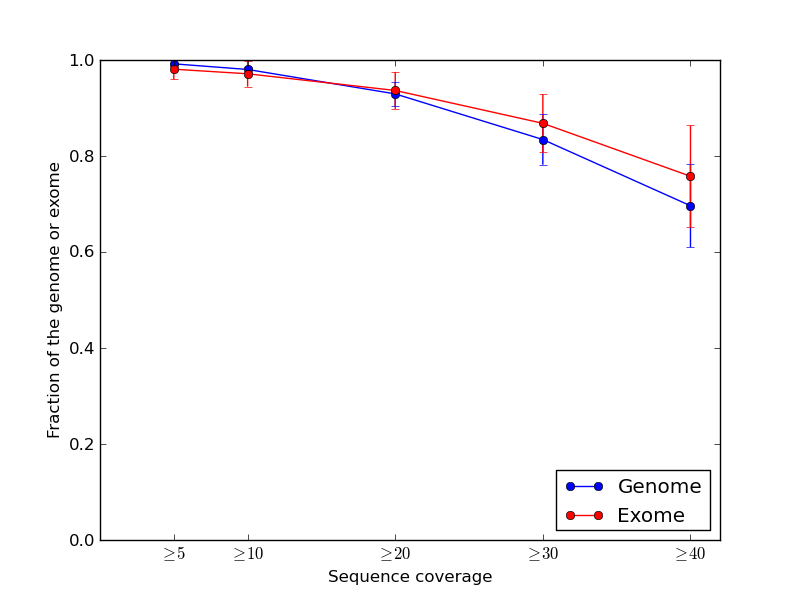

Supplement: Figure S2 — Weighted sequence coverage for the whole genome (blue) and exome (red) of WGS of 50 Amish samples. (PNG) [file pgen.1004229.s002.png]

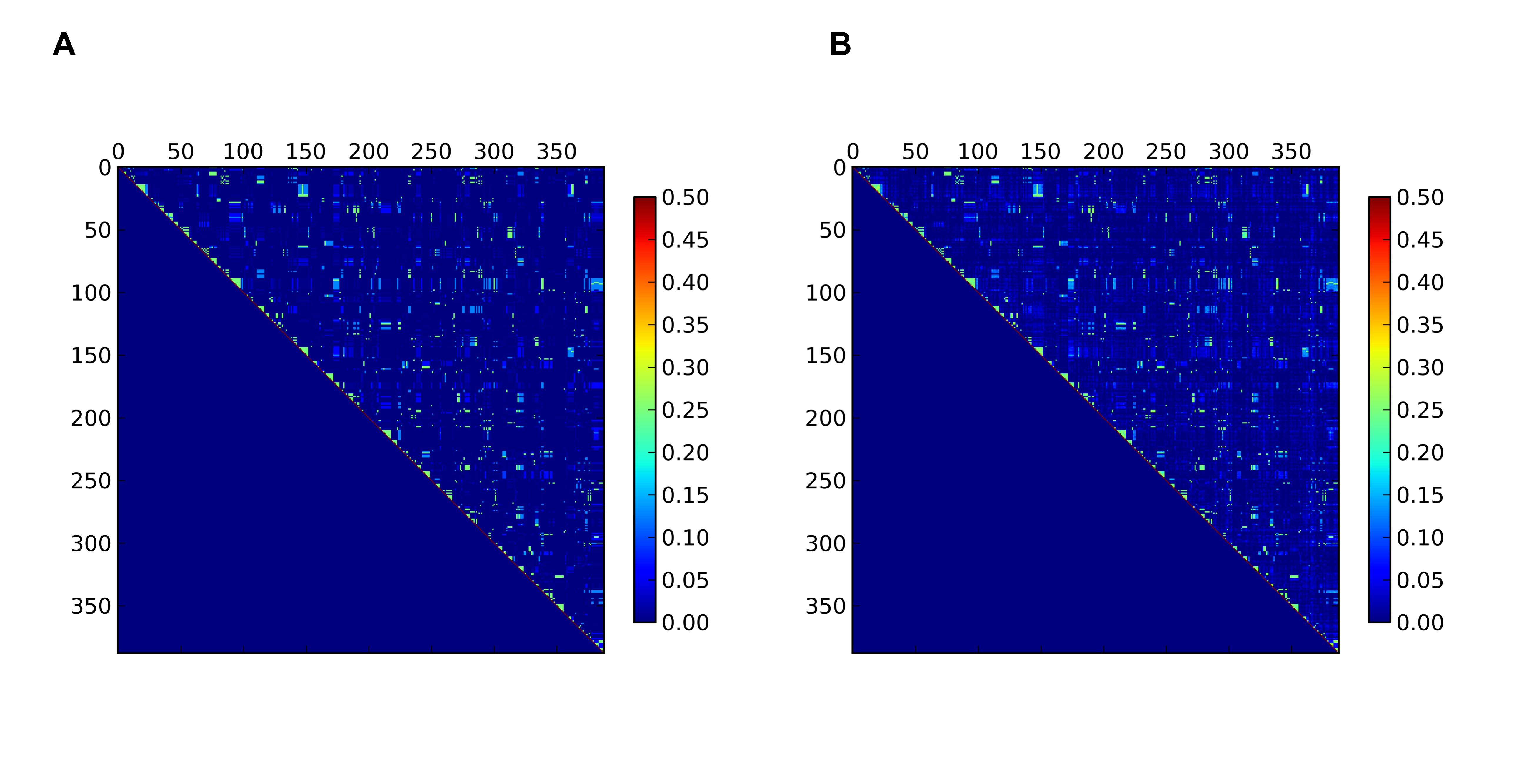

Supplement: Figure S3 — Pairwise kinship coefficient matrices for 388 samples with Omni2.5 SNP genotypes. A) Kinship coefficients based on known pedigree relationships. B) Estimated kinships coefficients based on 1.3M SNP genotypes. (TIF) [file pgen.1004229.s003.tif]

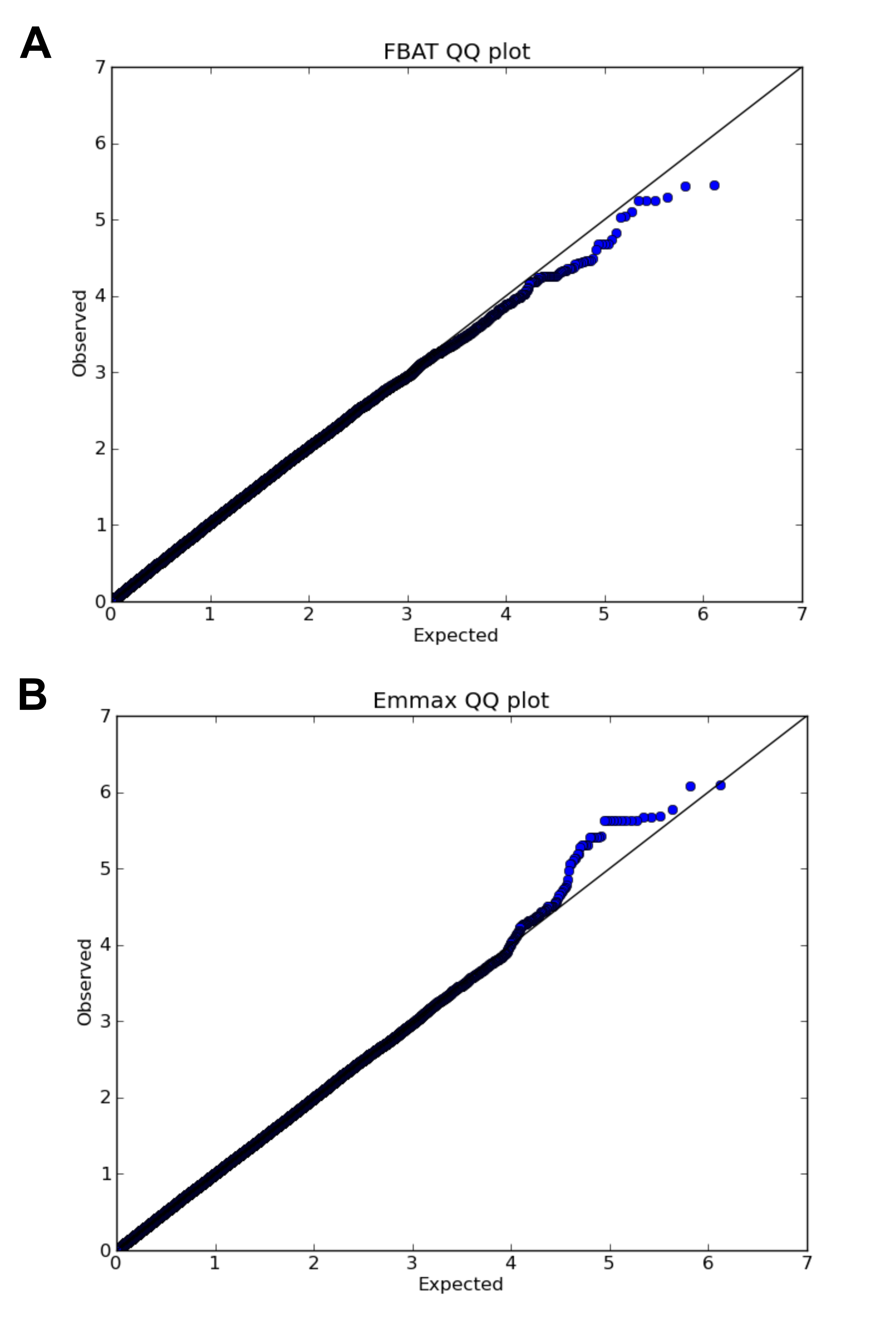

Supplement: Figure S4 — Quantile-Quantile plots of genome-wide association analysis. A) FBAT analysis and B) EMMAX analysis. (TIF) [file pgen.1004229.s004.tif]

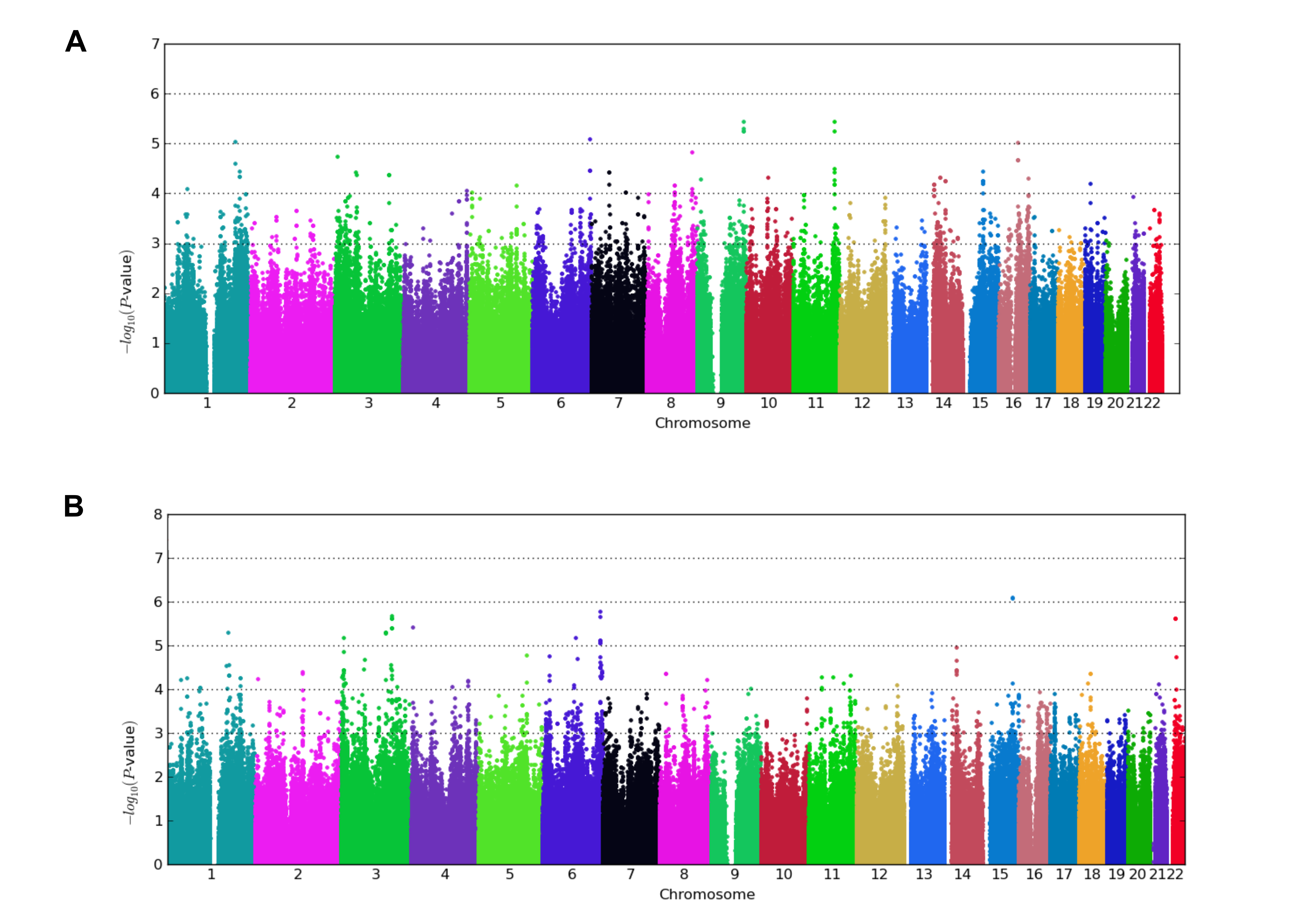

Supplement: Figure S5 — Manhattan plots of genome-wide association analysis of 388 samples from the extended Amish pedigree. A) Family-based association analysis using FBAT, B) Mixed model case-control analysis with correction for relatedness using EMMAX. (TIF) [file pgen.1004229.s005.tif]

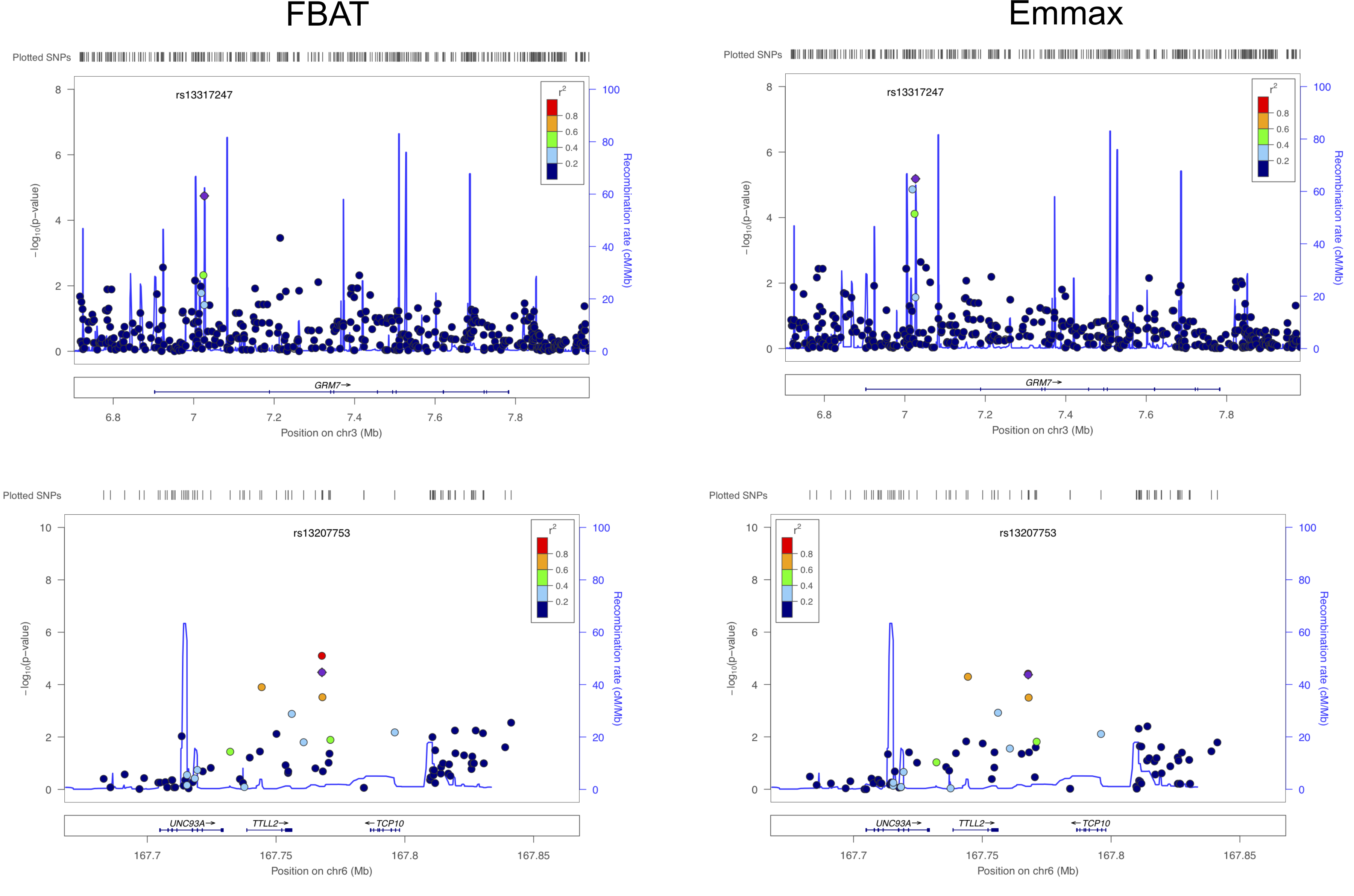

Supplement: Figure S6 — FBAT (left) and EMMAX (right) association results at the GRM7 (top) and TTLL2-TCP10 loci (bottom) (plots generated with LocusZoom). (TIF) [file pgen.1004229.s006.tif]

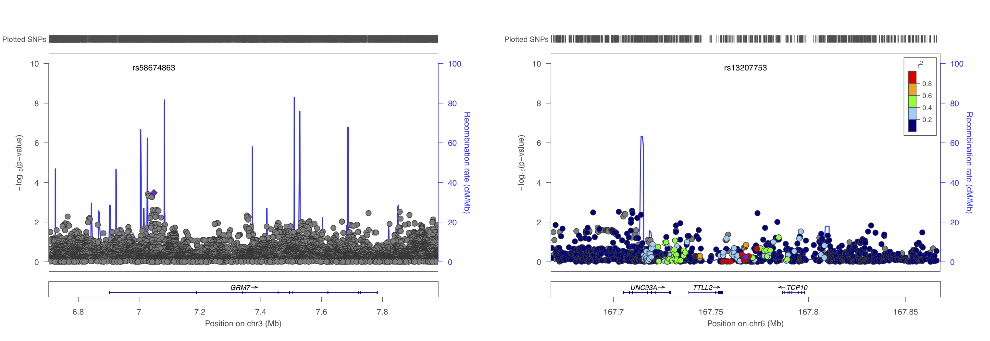

Supplement: Figure S7 — LocusZoom plots of case-control association for 2,836 BP cases and 2,744 controls (GAIN) at the GRM7 (left) and TTLL2-TCP10 (right) loci. (PNG) [file pgen.1004229.s007.png]

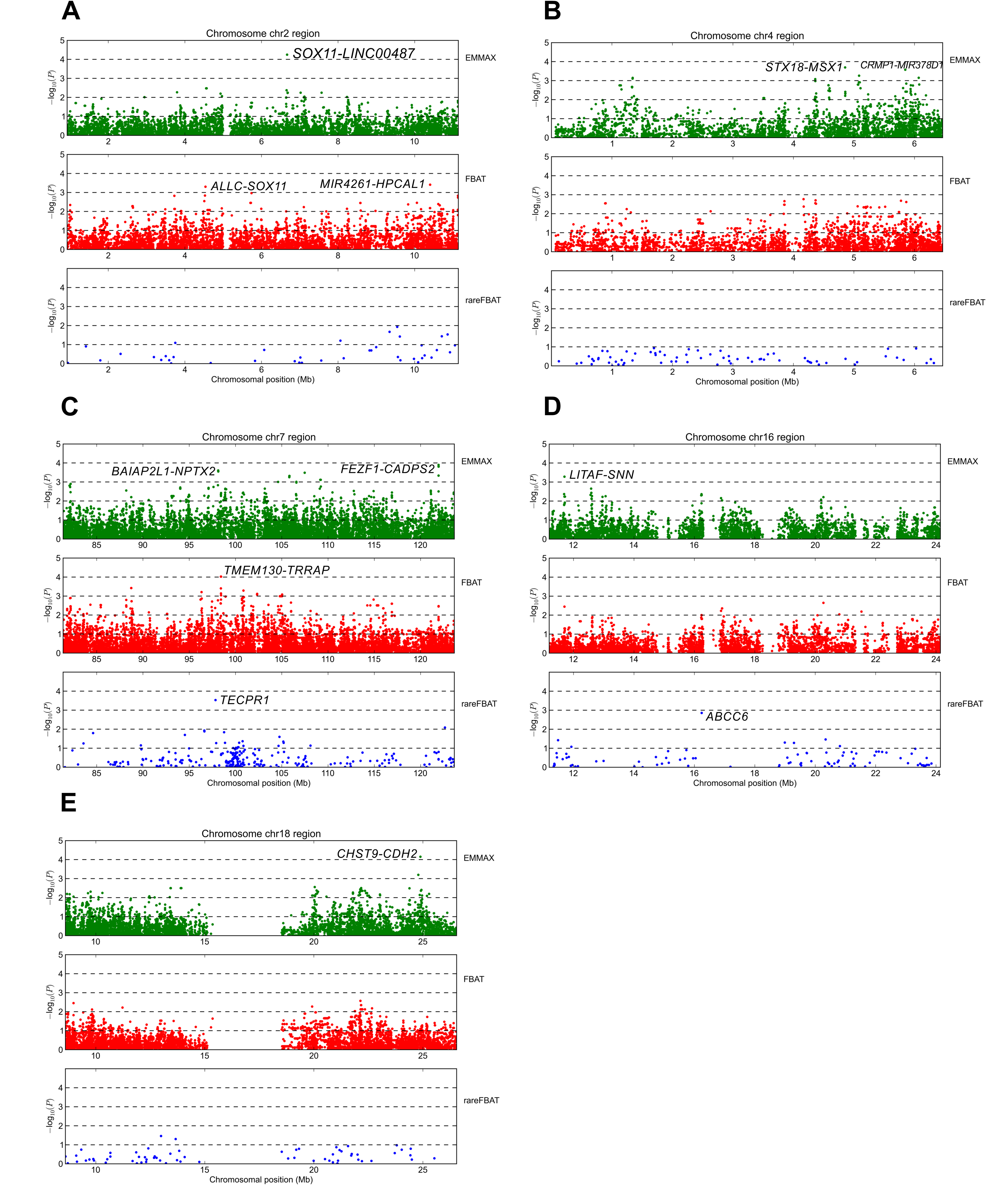

Supplement: Figure S8 — SNP-based and gene-wise burden association tests in the linkage regions. For each of the five identified linkage regions (A) 2p25, B) 4p16.3, C) 7q21, D) 16p13 and E) 18p11) association results with EMMAX and FBAT using Omni2.5 SNP array data (top and middle panel) are shown. The bottom panels display the results of gene-wise burden tests of exonic and regulatory variants using the rareFBAT test based on imputed WGS and Omni2.5 genotype data. The x-axis shows the chromosomal position in Mb, the y-axis the -log(P-values) for the respective method. Each data point corresponds to a single SNP in the EMMAX and FBAT panels and a combined burden test for exonic and regulatory variants in a single gene in the rare FBAT panels. (TIF) [file pgen.1004229.s008.tif]

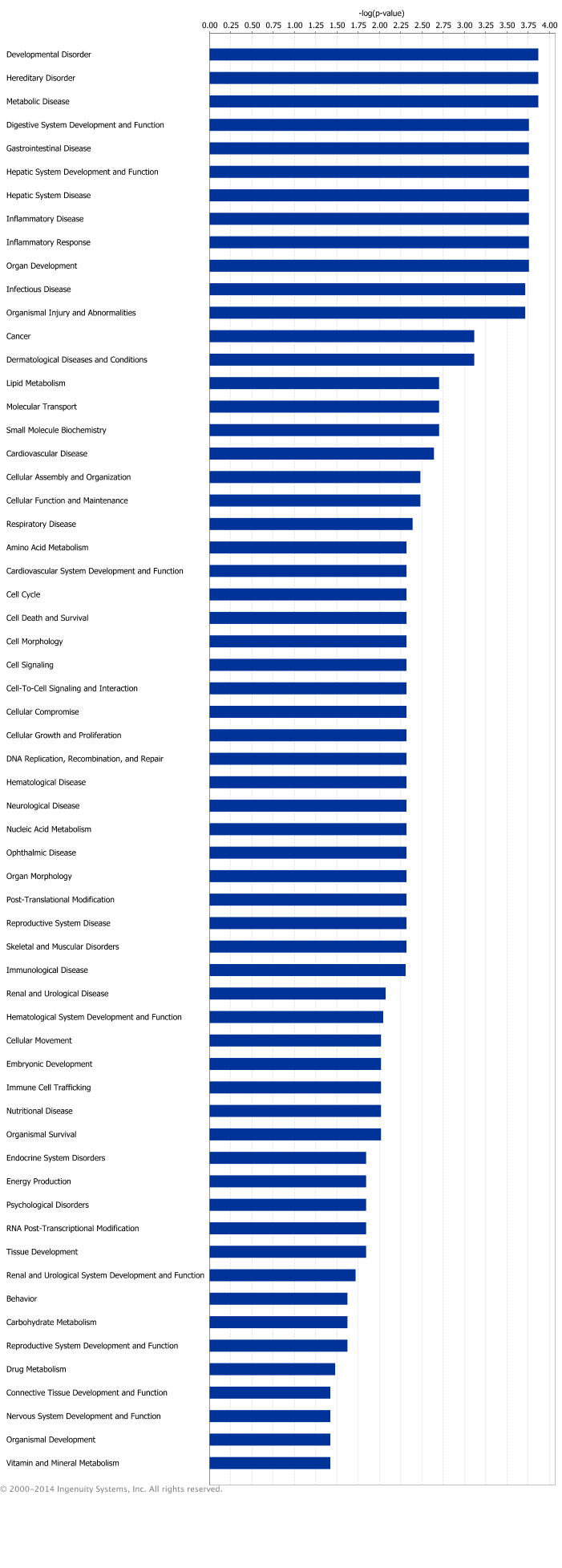

Supplement: Figure S9 — Example of an Ingenuity Pathway Analysis (IPA) of the top 100 genes from table S1. No significant enrichment for a specific pathway, biological function or tissue-specific gene expression was observed. (TIF) [file pgen.1004229.s009.tif]

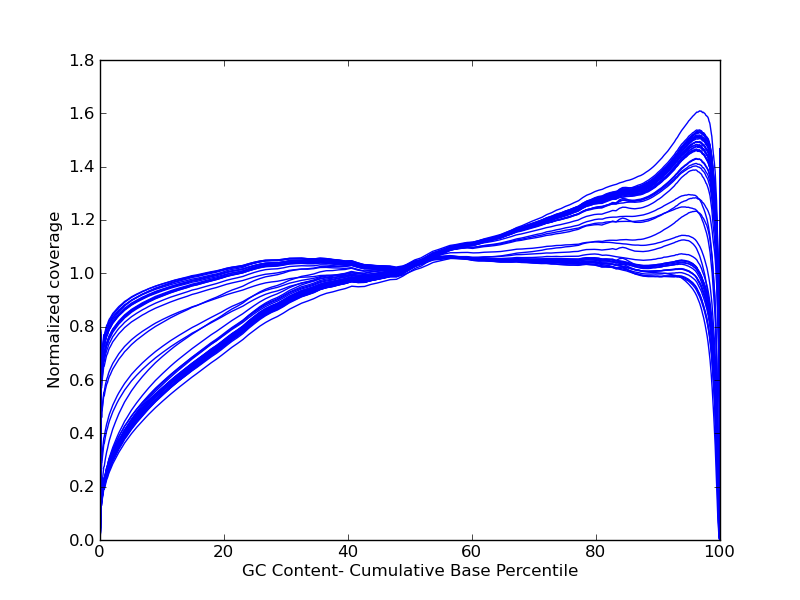

Supplement: Figure S10 — Normalized coverage (i.e. coverage relative to the genome average) for different GC content percentiles. GC content was calculated for bins of 501 bp across the genome. It can be seen that the normalized coverage only drops off at the very extremes of the GC content distribution in all 50 samples. (PNG) [file pgen.1004229.s010.png]

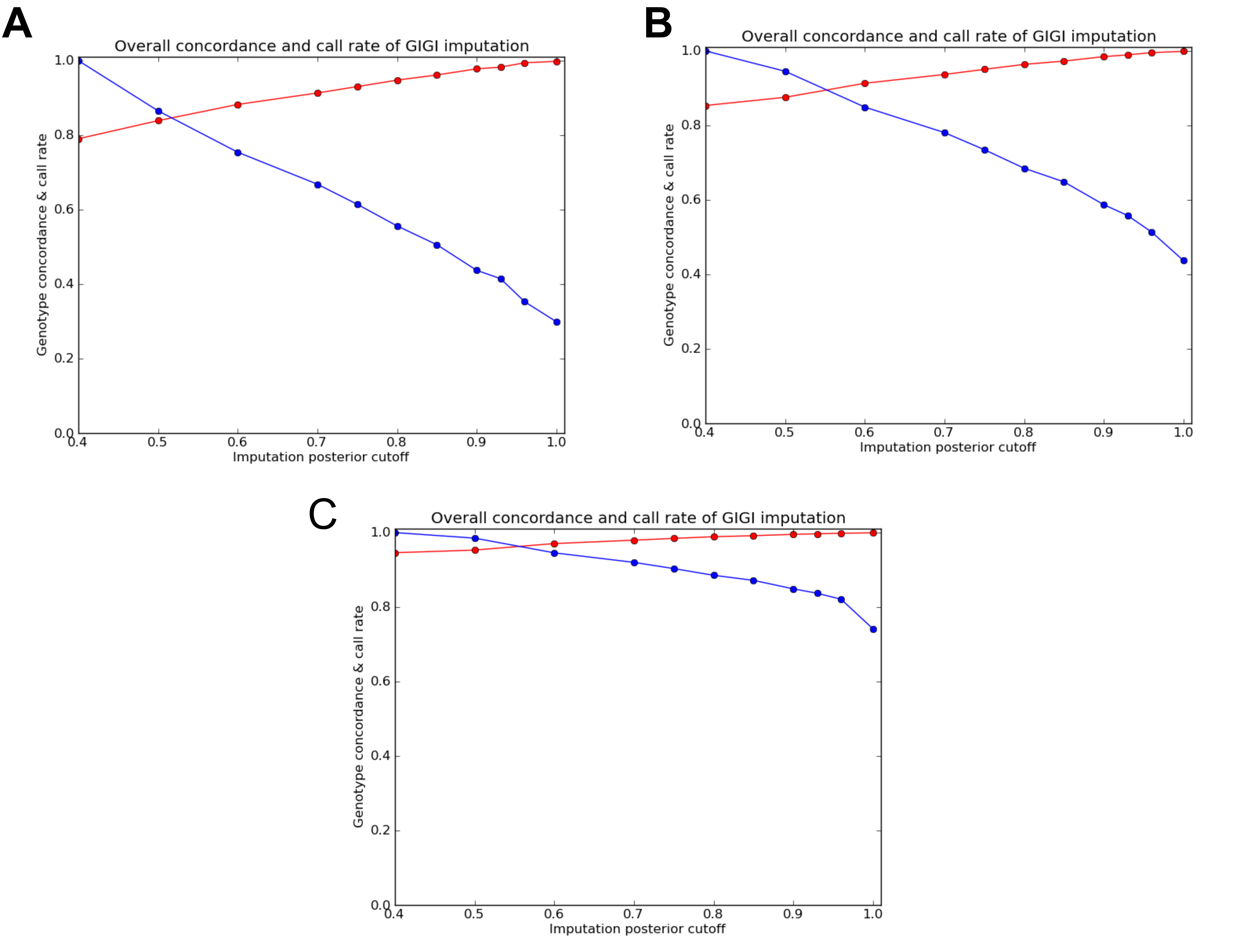

Supplement: Figure S11 — Evaluation of imputation performance for different thresholds on the genotype posterior probability. Concordance between imputed and SNP array genotypes (red) and call rate of the imputed genotypes (blue) are shown. The plot shows averages over A) all samples in the dataset, B) families in the neighborhood of WGS samples and C) first degree relatives of subjects with WGS. (TIF) [file pgen.1004229.s011.tif]
