# Supplementary material for: Genomic View of Bipolar Disorder Revealed by Whole Genome Sequencing in a Genetic Isolate
Source: PLoS Genet. 2014 Mar 13;10(3):e1004229. doi: 10.1371/journal.pgen.1004229 (PMC3953017; doi:10.1371/journal.pgen.1004229)
Supplement: Table S3 — Top SNPs from genome-wide association analysis with FBAT and EMMAX association analysis of 1.3M SNPs for 388 subjects from the extended pedigree. Only SNPs with p< = 5e-4 are shown. (DOCX) [file pgen.1004229.s014.docx]

| **Chrom** | **Position** | **Ref** | **Alt** | **Omni2.5 ID** | **Type** | **Gene** | **FBAT** | **EMMAX** |
| --- | --- | --- | --- | --- | --- | --- | --- | --- |
| 1 | 168873915 | G | A | kgp8093460 | intergenic | *LINC00626*-*ATP1B1* | 0.000473 | 0.000290072 |
| 1 | 168930428 | G | A | kgp1883447 | intergenic | *LINC00626*-*ATP1B1* | 0.000261 | 9.58E-05 |
| 1 | 168942578 | T | C | kgp2136511 | intergenic | *LINC00626*-*ATP1B1* | 0.000473 | 0.000290072 |
| 1 | 204877652 | G | A | rs2247208 | intronic | *NFASC* | 8.99E-06 | 0.000149282 |
| 1 | 204883953 | T | A | kgp10501567 | intronic | *NFASC* | 2.50E-05 | 1.32E-04 |
| 2 | 133733733 | C | A | kgp4433104 | intronic | *NCKAP5* | 0.000433 | 3.09E-04 |
| 2 | 133742448 | T | G | rs2320399 | intronic | *NCKAP5* | 0.00022 | 4.40E-05 |
| 2 | 133746273 | T | C | rs4954014 | intronic | *NCKAP5* | 0.000433 | 0.000469191 |
| 2 | 133746630 | A | C | kgp5005493 | intronic | *NCKAP5* | 0.000222 | 0.000104491 |
| 2 | 133748769 | C | T | rs4954015 | intronic | *NCKAP5* | 0.000433 | 0.000469191 |
| 3 | 6428805 | G | A | kgp2135639 | intergenic | *MIR4790*-*GRM7* | 0.000307 | 0.000178519 |
| 3 | 7026535 | T | C | rs13317247 | intronic | *GRM7* | 1.80E-05 | 6.54E-06 |
| 3 | 13924847 | G | A | kgp7362739 | intergenic | *WNT7A*-*LOC100132526* | 3.01E-04 | 2.62E-04 |
| 3 | 155458211 | G | A | kgp7914486 | intergenic | *PLCH1*-*C3orf33* | 4.30E-05 | 0.000167226 |
| 3 | 155470585 | C | A | rs6765861 | intergenic | *PLCH1*-*C3orf33* | 4.30E-05 | 1.79E-04 |
| 3 | 155490066 | G | T | rs17349490 | intronic | *C3orf33* | 4.30E-05 | 0.000178919 |
| 3 | 155490618 | C | T | kgp3064551 | intronic | *C3orf33* | 4.30E-05 | 0.000178919 |
| 3 | 155520717 | C | T | rs404570 | intronic | *C3orf33* | 1.51E-04 | 0.000270714 |
| 4 | 139155804 | C | T | rs7685529 | intronic | *SLC7A11* | 0.000244 | 0.000393953 |
| 6 | 167323997 | T | C | kgp3873204 | intergenic | *RPS6KA2*-*RNASET2* | 0.000438 | 4.86E-04 |
| 6 | 167744409 | C | T | rs6908815 | intronic | *TTLL2* | 1.25E-04 | 5.07E-05 |
| 6 | 167758095 | G | A | kgp5257373 | intergenic | *TTLL2-*,*TCP10* | 3.40E-05 | 4.23E-05 |
| 6 | 167767774 | C | T | rs13193386 | intergenic | *TTLL2-*,*TCP10* | 7.94E-06 | 3.85E-05 |
| 6 | 167767813 | T | G | rs13207753 | intergenic | *TTLL2-*,*TCP10* | 3.40E-05 | 4.23E-05 |
| 6 | 167767876 | C | T | kgp7112091 | intergenic | *TTLL2-*,*TCP10* | 0.000302 | 3.17E-04 |
| 6 | 167767987 | A | G | rs2981984 | intergenic | *TTLL2-*,*TCP10* | 0.000302 | 0.000316668 |
| 6 | 167769405 | T | C | kgp2498889 | intergenic | *TTLL2-*,*TCP10* | 3.40E-05 | 4.23E-05 |
| 8 | 73736766 | A | C | rs1107217 | intronic | *KCNB2* | 3.44E-04 | 4.90E-04 |
| 12 | 30925894 | C | T | rs11051070 | intergenic | *CAPRIN2*-*LOC100287314* | 3.05E-04 | 2.76E-04 |
| 14 | 33785548 | T | C | rs1952302 | intronic | *NPAS3* | 4.05E-04 | 1.07E-05 |
| 15 | 91593843 | G | A | kgp6481414 | intergenic | *VPS33B*-*SV2B* | 3.10E-04 | 1.96E-04 |
| 16 | 55880480 | C | A | kgp12493768 | exonic | *CES5A* | 2.10E-05 | 0.000396122 |
| 16 | 55880639 | T | C | kgp7914703 | intronic | *CES5A* | 2.10E-05 | 0.000396122 |
| 16 | 55880825 | G | T | kgp265799 | intronic | *CES5A* | 2.10E-05 | 0.000396122 |
| 16 | 55880874 | A | G | rs11076122 | intronic | *CES5A* | 2.10E-05 | 0.000396122 |
| 16 | 57672186 | A | C | rs12446402 | intronic | *GPR56* | 0.000494 | 0.000269586 |
| 16 | 60404842 | T | C | kgp7698768 | intergenic | *LOC644649*-*CDH8* | 0.000325 | 0.00011318 |
| 16 | 79510358 | G | A | rs11642762 | intergenic | *WWOX*-*MAF* | 3.35E-04 | 0.00036545 |
| 16 | 79511303 | G | A | kgp6947644 | intergenic | *WWOX*-*MAF* | 0.000355 | 0.000416949 |
| 16 | 79512420 | G | A | kgp5034622 | intergenic | *WWOX*-*MAF* | 3.35E-04 | 0.00036545 |
| 16 | 79513098 | A | G | rs11150167 | intergenic | *WWOX*-*MAF* | 0.000335 | 0.000285522 |
| 16 | 84575602 | A | C | rs8056021 | intergenic | *KIAA1609*-*COTL1* | 0.000105 | 0.000359196 |
