# Supplementary material for: Genomic View of Bipolar Disorder Revealed by Whole Genome Sequencing in a Genetic Isolate
Source: PLoS Genet. 2014 Mar 13;10(3):e1004229. doi: 10.1371/journal.pgen.1004229 (PMC3953017; doi:10.1371/journal.pgen.1004229)
Supplement: Table S6 — A) Linkage results from the analysis of 49 nuclear families from the extended pedigree and B) linkage results from the analysis of defined subpedigrees. The table lists the chromosomal region, top LOD marker, length of peak region for a 2 LOD confidence interval, phenotype under which maximum linkage was observed, linear non-parametric LOD score (NPL LOD), exponential non-parametric LOD score (NPL expLOD), parametric LOD score, parametric HLOD score and the alpha value denoting the fraction of linked families in the HLOD computations. (DOC) [file pgen.1004229.s017.doc]

| Chrom. location | Marker  (bp) | Peak  length (Mb) | Phenotype | NPL LOD | NPL  expLOD | Param LOD  (model) | param. HLOD  (model) | alpha |
| --- | --- | --- | --- | --- | --- | --- | --- | --- |
| 1p36.22-p36.11 | *D1S2672*  (15278655) | 13.8 | BPS | 2.26 | 1.55 | <0.0  (dom 0.5) | 0.319  (rec 0.5) | 0.177 |
| 4p16.3-p16.1 | *D4S3023*  (4301334) | 8.1 | BPS | 0.53 | 0.68 | <0.0  (rec 0.5) | 2.347  (rec 0.5) | 0.357 |
| 7q21.11-q31.33 | *D7S518*  (101862217) | 42.3 | BPS | 2.99 | 2.55 | <0.0  (dom 0.5) | 0.220  (dom 0.5) | 0.179 |
| 10q23.33-q26.2 | *D10S1701*  (121184529) | 32.6 | BPS | 1.67 | 1.3 | 0.734  (dom 0.5) | 2.227  (dom 0.5) | 0.624 |
| 16p13.13-13.12 | *D16S3075*  (12209197) | 1.7 | BPS | 0.82 | 1.22 | <0.0  (rec 0.5) | 2.795  (rec 0.5) | 0.365 |
| 18p11.22-q12.1 | *D18S453*  (12914782) | 19.7 | BPI | 2.43 | 2.76 | 1.978  (dom 0.5) | 2.067  (dom 0.5) | 0.824 |

**A)**

**B)**

| Data | Chrom. location | Marker (bp) | Peak length  (Mb) | Phenotype | NPL LOD | NPL  expLOD | param LOD  (model) | param. HLOD  (model) | alpha |
| --- | --- | --- | --- | --- | --- | --- | --- | --- | --- |
| NB7 | 13q14.11-q33.3 | *D13S1816*  (88637566) | 63.7 | BPI | 0.3 | 0.6 | 2.371  (dom .85) | 2.371  (dom .85) | 1 |
| NB6 | 9q31.3 | *D9S1776*  (117959422) | 27.7 | BPI | 0.44 | 0.62 | 2.217  (rec 0.85) | 2.221  (rec 0.85) | 0.94 |
| NB6 | 2p25.3-p25.1 | *D2S2211*  (7471377) | 10.3 | Narrow | 1.14 | 3.01 | 1.492  (rec 0.5) | 1.492  (rec 0.5) | 1 |
| NB6 | 4p16.3-p16.1 | *D4S3360*  (115904) | 6.3 | Narrow | 0.84 | 1.67 | 3.959  (rec 0.85) | 3.959  (rec .85) | 1 |
| NB4 | 16p13.13-p12.1 | *D16S3127*  (15870863) | 12.9 | Narrow | 1.27 | 3.15 | 2.333  (rec 0.5) | 2.333  (rec 0.5) | 1 |
| NB4 | 2q21.1-q31.1 | *D2S2196*  (136888654) | 41.5 | BPI | 0.97 | 2.23 | 1.664  (rec 0.5) | 1.664  (rec 0.5) | 1 |
| NB4 | 10q25.2-q26.13 | *D10S1693*  (119444836) | 13.3 | BPS | 1.06 | 1.51 | 2.724  (dom 0.5) | 2.724  (dom 0.5) | 1 |
| NB3 | 22q11.22-q13.1 | *D22S315*  (26015839) | 17.6 | BPS | 0.6 | 0.25 | 2.806  (dom .85) | 2.806  (dom .85) | 1 |
| NB3 | 6q16.3-q22.31 | *D6S416* (112451276) | 23.7 | BPS | 1.2 | 2.41 | 2.705  (rec 0.85) | 2.705  (rec .85) | 1 |
| NB1 | 5q35.1-q35.3 | *D5S469*  (176770544) | 10.2 | Narrow | 0.47 | 0.39 | 2.305  (rec 0.85) | 2.305  (rec .85) | 1 |
| NB1 | 14q11.2-q23.1 | *D14S283*  (22687414) | 35.1 | Narrow | 1.16 | 2.42 | 1.556  (dom 0.5) | 1.556  (dom 0.5) | 1 |
