# Supplementary material for: Genomic View of Bipolar Disorder Revealed by Whole Genome Sequencing in a Genetic Isolate
Source: PLoS Genet. 2014 Mar 13;10(3):e1004229. doi: 10.1371/journal.pgen.1004229 (PMC3953017; doi:10.1371/journal.pgen.1004229)
Supplement: Table S7 — Number of linked families and contributing haplotypes for five identified linkage regions. (DOC) [file pgen.1004229.s018.doc]

| **Data set** | **Chromosome** | **Markers around top linkage** | **Number of linked families** | **Number of haplotypes (unique to one family)** |
| --- | --- | --- | --- | --- |
| All | chr7 | *D7S554, D7S518, D7S2509, D7S1530* | 16 | 28 (23) |
| All | chr18 | *D18S53, D18S453, DG18S9, D18S869* | 9 | 17 (14) |
| NB6 | chr2 | *D2S330, D2S2211, D2S1329, D2S162* | 3 | 4 (2) |
| NB6 | chr4 | *D4S3360, D4S2936, D4S412* | 3 | 4 (2) |
| NB4 | chr16 | *D16S500, D16S3127, D16S501, D16S410* | 4 | 7 (7) |
