# Supplementary material for: Genomic View of Bipolar Disorder Revealed by Whole Genome Sequencing in a Genetic Isolate
Source: PLoS Genet. 2014 Mar 13;10(3):e1004229. doi: 10.1371/journal.pgen.1004229 (PMC3953017; doi:10.1371/journal.pgen.1004229)
Supplement: Table S8 — FBAT haplotype (D4S3360, D4S2936, D4S412) association test results for linked families in the 4p16 linkage region under an additive model. Haplotypes identified by allele sharing in affected siblings are shown in bold. Significant haplotype association (p = 0.0267) is observed for the 5-3-2 haplotype. The global FBAT haplotype association test also was significant with p = 0.0291. (DOC) [file pgen.1004229.s019.doc]

| **Haplotypes** | **afreq** | **fam#** | **Z** | **P** |
| --- | --- | --- | --- | --- |
| **5-3-2** | 0.188 | 3 | 2.216 | 0.026716 |
| 1-1-5 | 0.128 | 1 | -1.414 | 0.157299 |
| **5-4-6** | 0.125 | 2 | 1.74 | 0.081904 |
| 2-7-4 | 0.063 | 1 | -1.414 | 0.157299 |
| **4-2-2** | 0.063 | 1 | 1.414 | 0.157299 |
| 2-7-6 | 0.063 | 1 | -1.414 | 0.157299 |
| **4-5-5** | 0.063 | 1 | 1.414 | 0.157299 |
| 2-3-6 | 0.063 | 1 | -2.012 | 0.044234 |
| 4-3-5 | 0.063 | 1 | -2 | 0.0455 |
