# Supplementary material for: Genomic View of Bipolar Disorder Revealed by Whole Genome Sequencing in a Genetic Isolate
Source: PLoS Genet. 2014 Mar 13;10(3):e1004229. doi: 10.1371/journal.pgen.1004229 (PMC3953017; doi:10.1371/journal.pgen.1004229)
Supplement: Table S9 — Genes within the five linkage regions with nominally significant burden test association (P<0.05). The table shows the peak region, Gene, number of exonic and regulatory SNPs included in the burden test and rareFBAT P-values for the Null of hypotheses of “No linkage, no association” and “linkage and no association”. (DOCX) [file pgen.1004229.s020.docx]

| **Peak region** | **Gene** | **#SNPs** | **rFBAT P** | **corr. rFBAT P** |
| --- | --- | --- | --- | --- |
| 2p25 | *ITGB1BP1* | 143 SNPs | 0.005225539 | 0.011692966 |
| 2p25 | *ASAP2* | 96 SNPs | 0.012285286 | 0.021529858 |
| 2p25 | *IAH1* | 106 SNPs | 0.026574141 | 0.037795409 |
| 2p25 | *LOC339788* | 9 SNPs | 0.02937403 | 0.062645029 |
| 7q21 | *TECPR1* | 255 SNPs | 2.08E-05 | 0.000295294 |
| 7q21 | *PPP1R9A* | 27 SNPs | 0.002827008 | 0.020239853 |
| 7q21 | *DLX6* | 7 SNPs | 0.003098982 | 0.011832154 |
| 7q21 | *RINT1* | 88 SNPs | 0.004830505 | 0.045110033 |
| 7q21 | *KPNA7* | 2 SNPs | 0.005831539 | 0.014478561 |
| 7q21 | *SERPINE1* | 9 SNPs | 0.006455638 | 0.043325772 |
| 7q21 | *STEAP1* | 320 SNPs | 0.006787847 | 0.071277603 |
| 7q21 | *EFCAB10* | 48 SNPs | 0.007712646 | 0.052303198 |
| 7q21 | *DLX5* | 30 SNPs | 0.008206635 | 0.01317243 |
| 7q21 | *TAS2R16* | 5 SNPs | 0.014225836 | 0.008214055 |
| 7q21 | *MLL5* | 80 SNPs | 0.014691906 | 0.076013981 |
| 7q21 | *PON3* | 64 SNPs | 0.015987476 | 0.102891246 |
| 7q21 | *ZNHIT1* | 36 SNPs | 0.018945123 | 0.075786189 |
| 7q21 | *SEMA3D* | 13 SNPs | 0.019292943 | 0.016046758 |
| 7q21 | *LOC100216546* | 78 SNPs | 0.019922818 | 0.169742859 |
| 7q21 | *EPHB4* | 4 SNPs | 0.020764974 | 0.051792365 |
| 7q21 | *LRRC17* | 16 SNPs | 0.021952156 | 0.125439184 |
| 7q21 | *SRPK2* | 5 SNPs | 0.022036277 | 0.025425058 |
| 7q21 | *BAIAP2L1* | 116 SNPs | 0.02295219 | 0.05407498 |
| 7q21 | *EMID2* | 120 SNPs | 0.027592961 | 0.121110737 |
| 7q21 | *ZAN* | 27 SNPs | 0.033673153 | 0.089128422 |
| 7q21 | *C7orf61* | 15 SNPs | 0.034212458 | 0.095834721 |
| 7q21 | *PILRA* | 146 SNPs | 0.036598944 | 0.11616126 |
| 7q21 | *MYH16* | 2 SNPs | 0.038070618 | 0.052397841 |
| 7q21 | *GIGYF1* | 57 SNPs | 0.04016758 | 0.089731239 |
| 16p13 | *ABCC6* | 5 SNPs | 0.000553598 | 0.001400696 |
| 16p13 | *CLEC19A* | 1 SNPs | 0.012688576 | 0.052656311 |
| 16p13 | *SNN* | 36 SNPs | 0.027084713 | 0.198154562 |
| 16p13 | *RSL1D1* | 24 SNPs | 0.029803761 | 0.084017859 |
| 16p13 | *SCNN1B* | 25 SNPs | 0.038912767 | 0.107440316 |
| 16p13 | *TMC7* | 1 SNPs | 0.040377248 | 0.050347975 |
| 18p11 | *C18orf19* | 215 SNPs | 0.009406328 | 0.050191313 |
| 18p11 | *CEP192* | 222 SNPs | 0.011337638 | 0.034545624 |
| 18p11 | *CCDC165* | 53 SNPs | 0.024027639 | 0.063404981 |
| 18p11 | *PTPRM* | 22 SNPs | 0.02970108 | 0.071264482 |
| 18p11 | *LAMA3* | 85 SNPs | 0.031897575 | 0.221190332 |
| 18p11 | *SNRPD1* | 68 SNPs | 0.035758768 | 0.181864087 |
| 18p11 | *MIB1* | 54 SNPs | 0.040700449 | 0.163753166 |
