# Supplementary material for: Genomic View of Bipolar Disorder Revealed by Whole Genome Sequencing in a Genetic Isolate
Source: PLoS Genet. 2014 Mar 13;10(3):e1004229. doi: 10.1371/journal.pgen.1004229 (PMC3953017; doi:10.1371/journal.pgen.1004229)
Supplement: Table S15 — Numbers of families and subjects with WGS data for the entire pedigree and seven more homogeneous subpedigrees chosen based on family relationship and imputation quality. (DOC) [file pgen.1004229.s026.doc]

| **Neighborhood** | **# Families** | **# WGS samples** | **Pedigree branch** |
| --- | --- | --- | --- |
| All | 49 | 50 | - |
| NB1 | 9 | 13 | 110C |
| NB2 | 2 | 3 | 110R |
| NB3 | 5 | 7 | 410 |
| NB4 | 7 | 11 | 310 |
| NB5 | 3 | 1 | 210/410 |
| NB6 | 5 | 9 | 310 |
| NB7 | 3 | 7 | 410/310 |
